# The C-Terminal Acidic Tail Modulates the Anticancer Properties of HMGB1

**DOI:** 10.3390/ijms23147865

**Published:** 2022-07-17

**Authors:** Chloé Borde, Clémentine Dillard, Aurore L’Honoré, Frédérique Quignon, Marion Hamon, Christophe H. Marchand, Roberta Soares Faccion, Maurício G. S. Costa, Elodie Pramil, Annette K. Larsen, Michèle Sabbah, Stéphane D. Lemaire, Vincent Maréchal, Alexandre E. Escargueil

**Affiliations:** 1 Sorbonne Université, Institut National de la Santé et de la Recherche Médicale (INSERM) U938, Centre de Recherche Saint-Antoine, F-75012 Paris, France; chloe.borde_chivot@sorbonne-universite.fr (C.B.); clementinedillard@gmail.com (C.D.); robsfaccion@gmail.com (R.S.F.); elodie.pramil@hotmail.fr (E.P.); annettelarsen004@gmail.com (A.K.L.); michele.sabbah@inserm.fr (M.S.); 2 Sorbonne Université, Centre National de la Recherche Scientifique (CNRS), INSERM, Institut de Biologie Paris-Seine, Biological Adaptation and Aging, B2A-IBPS, F-75005 Paris, France; aurore.lhonore@sorbonne-universite.fr; 3 Sorbonne Université, CNRS UMR 144, Institut Curie Centre de Recherche, F-75248 Paris, France; frederique.quignon@sorbonne-universite.fr; 4 Centre National de la Recherche Scientifique (CNRS), Institut de Biologie Physico-Chimique, Plateforme de Protéomique, FR550, F-75005 Paris, France; marion.hamon@ibpc.fr (M.H.); christophe.marchand@sorbonne-universite.fr (C.H.M.); 5 Sorbonne Université, Centre National de la Recherche Scientifique (CNRS), Institut de Biologie Paris-Seine, UMR7238, Laboratory of Computational and Quantitative Biology, F-75005 Paris, France; stephane.lemaire@sorbonne-universite.fr; 6 Sorbonne Université, Centre National de la Recherche Scientifique (CNRS), Institut de Biologie Physico-Chimique, UMR8226, F-75005 Paris, France; 7 Laboratório de Hemato-Oncologia Celular e Molecular, Programa de Hemato-Oncologia Molecular, Hospital do Câncer I, Centro de Pesquisas do Instituto Nacional de Câncer José Alencar Gomes da Silva (INCA), Praça da Cruz Vermelha 23/6° andar, Rio de Janeiro 20230-130, Brazil; 8 Fundação Oswaldo Cruz, Programa de Computação Científica, Vice-Presidência de Educação, Informação e Comunicação, Av. Brasil 4365, Manguinhos, Rio de Janeiro 21040-900, Brazil; mauricio.costa@fiocruz.br; 9 Alliance for Research in Cancerology-APREC, Tenon Hospital, F-75020 Paris, France

**Keywords:** HMGB1, anticancer agent, cell bioenergetics, pyruvate kinase

## Abstract

Energy metabolism reprogramming was recently listed as a hallmark of cancer. In this process, the switch from pyruvate kinase isoenzyme type M1 to pyruvate kinase isoenzyme type M2 (PKM2) is believed to play a crucial role. Interestingly, the activity of the active form of PKM2 can efficiently be inhibited by the high-mobility group box 1 (HMGB1) protein, leading to a rapid blockage of glucose-dependent aerobic respiration and cancer cell death. HMGB1 is a member of the HMG protein family. It contains two DNA-binding HMG-box domains and an acidic C-terminal tail capable of positively or negatively modulating its biological properties. In this work, we report that the deletion of the C-terminal tail of HMGB1 increases its activity towards a large panel of cancer cells without affecting the viability of normal immortalized fibroblasts. Moreover, in silico analysis suggests that the truncated form of HMGB1 retains the capacity of the full-length protein to interact with PKM2. However, based on the capacity of the cells to circumvent oxidative phosphorylation inhibition, we were able to identify either a cytotoxic or cytostatic effect of the proteins. Together, our study provides new insights in the characterization of the anticancer activity of HMGB1.

## 1. Introduction

Cancer is an evolutionary-driven disease that is characterized by the ability of tumor cells to adapt to a wide range of environmental stresses [1,2]. This leads to the selection of phenotypes whose aggressiveness usually increases with the stage of cancer [3]. In the last decades, the reprogramming of the energy metabolism was listed as an emerging hallmark of cancer [4,5]. Under aerobic conditions, normal differentiated cells usually extract energy from glucose mostly through oxidative phosphorylation. In contrast, tumor cells are believed to preferentially metabolize glucose to lactate even in the presence of oxygen [4], a process known as the Warburg effect. In these processes, the pyruvate kinase (PK) isozymes play a crucial role. PK is a rate limiting glycolytic enzyme that converts phosphoenolpyruvate and ADP to pyruvate and ATP. In humans, four pyruvate kinase isoforms can be found: (1) PKL, which is primarily expressed in the liver and kidneys; (2) PKR, which is exclusively expressed in red blood cells; (3) PKM1 (pyruvate kinase isoenzyme type M1), which is highly expressed in differentiated tissues with high energetic demands; and (4) PKM2 (pyruvate kinase isoenzyme type M2), which is highly expressed in undifferentiated tissues as well as in rapidly proliferating tissues including cancer [6]. PKM1 and PKM2 are produced by alternative splicing of transcripts expressed from the same *PKM* gene. PKM1 and PKM2 differ in the inclusion of exon 9 or 10. PKM1 exclusively contains exon 9 whereas PKM2 exclusively contains exon 10. Isoform switch from PKM1 to PKM2 and elevated expression of PKM2 have been reported in many types of cancers including glioblastoma, colon and breast cancers [7,8,9,10,11,12,13,14] and is associated with poor prognosis in head and neck cancers, signet ring cell gastric cancer and esophageal squamous cell cancer [11,15,16]. Interestingly, it has been reported that the activity of the tetrameric form of PKM2 can be efficiently inhibited allosterically by the phosphorylated form of the high-mobility group box 1 (HMGB1) protein, leading to a rapid blockage of glucose-dependent aerobic respiration and cancer cell death making PKM2 an attractive therapeutic target [17,18].

HMGB1 is an evolutionary-conserved member of the HMG protein family and was first isolated and characterized from calf thymus [19]. In most cells, it is located in the nucleus, where it was initially found to act as a non-histone architectural chromatin-binding factor [19,20]. Later, HMGB1 was discovered to be expressed on cell surface membranes, cytosol and mitochondria and released into the extracellular space where it forms complexes with various molecules [21].

HMGB1 contains two DNA-binding domains, HMG-box A and B, located in the N-terminal and the central part of the protein, respectively, and an acidic C-terminal tail with numerous aspartic or glutamic acid residues. The A ([1–79] amino acid sequence) and B ([89–162] amino acid sequence) boxes interact with DNA to bend or distort the double helix [22,23]. The B box is responsible for the cytokine activity of HMGB1 and efficiently induces the secretion of additional pro-inflammatory cytokines by macrophages [24]. The B box is also involved in the specific binding to PKM2 [18]. Importantly, three highly conserved cysteine residues at positions 23, 45 and 106 are present in HMGB1 and are subject to redox modifications [22]. Various reports demonstrated that the activities of HMGB1 strongly depend on the redox state of these three cysteine residues [25]. In particular, these cysteines play a role in crucial HMGB1 extracellular functions by modulating its binding capacity to receptors and thus HMGB1 cytokine or chemoattractant activities [20]. In addition to the A and B boxes, the C-terminal tail ([186–215] amino acid sequence) of HMGB1 also plays an important role in its biological functions. It contributes to the spatial arrangement of both A and B boxes and modulates HMGB1 DNA-binding specificity [23,26,27,28]. Furthermore, the acidic tail has been shown to bind to the HMGB1 boxes in vitro [26,29,30,31], and this phenomenon is believed to negatively regulate HMGB1–DNA interactions and thus to modulate its DNA binding properties [31]. Although the C-terminal tail–boxes complex is a dynamic system, it has been proposed that because of its close proximity to the boxes, the C-terminal acidic tail can shield them from other interactions, thus affecting their biological functions [32]. In agreement, the C-terminal tail is important for the function of HMGB1 in the stimulation of transcription [33,34] and nucleosome remodeling [35]. The deletion of the C-terminal tail of HMGB1 is also capable of preventing the binding of HMGB1 to RAGE (receptor for advanced glycation end products) and to subsequently limit the inhibitory effects of HMGB1 on efferocytosis [36]. Intriguingly, the C-terminal acidic tail of HMGB1 has also been reported as playing a key role in the antibacterial activity of the protein. A truncated form of HMGB1 lacking its C-terminal acidic tail indeed failed to inhibit bacterial multiplication [37].

In this work, we investigated whether the deletion of the C-terminal tail of HMGB1 might affect its anticancer activity as well as its effect on the metabolism of cancer cells. Our results demonstrate that the deletion of the C-terminal tail of HMGB1 increases its activity towards a large panel of cancer cells including cells resistant to DNA-targeted chemotherapy without affecting the viability of normal immortalized fibroblasts. Moreover, the truncated form of HMGB1 (named hereafter as HMGB1-ΔC) retains the capacity of the full-length protein HMGB1-fl to interact with PKM2 and to perturb the metabolism of cancer cells. However, based on the capacity of the cells to circumvent oxidative phosphorylation inhibition, we were able to identify either a cytotoxic or cytostatic effect of both the full-length and truncated proteins. Together, our study provides new insights in the characterization of the anticancer activity of HMGB1.

## 2. Results

### 2.1. Production and Characterization of HMGB1-ΔC

**Figure 1:**
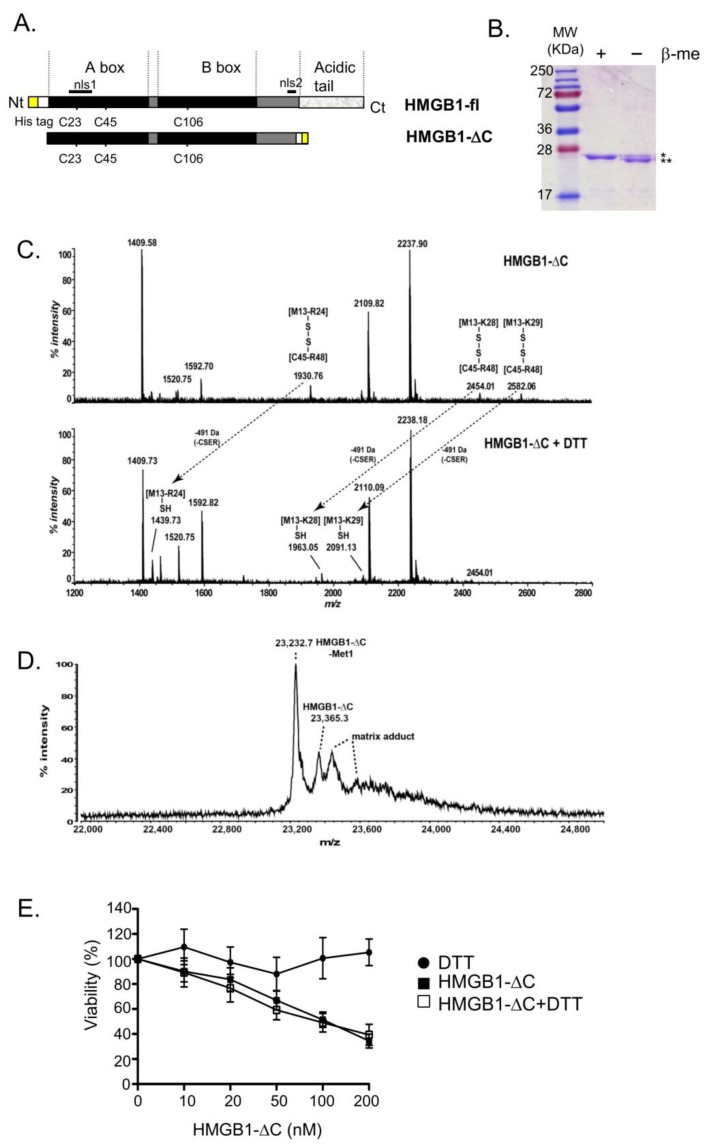
Production and characterization of HMGB1-ΔC. (**A**) Plasmid constructions of HMGB1-fl (pET15b-6His-HMGB1) and HMGB1-ΔC (pET-22b(pelB-)HMGB1-ΔC) are schematically illustrated. The pET15b-6His-HMGB1 expression vector encodes for HMGB1-fl fused to a hexa-histidine tag (yellow box) at its N-terminus. The pET-22b(pelB-)HMGB1-ΔC expression vector encodes for HMGB1-ΔC fused to a hexa-histidine tag at its C-terminus. (**B**) Expression and purification of HMGB1-ΔC. An amount of 1 µg of HMGB1-ΔC was analyzed by SDS-PAGE (12%) after Coomassie Blue staining. Before loading, fractions were boiled in a buffer containing (+β-me) or not (−β-me) 10% ß-mercaptoethanol (*: reduced HMGB1-ΔC, **: oxidized HMGB1-ΔC). (**C**) Peptide mass fingerprints analysis of the recombinant HMGB1-ΔC protein. Before analysis by MALDI-TOF mass spectrometry, the peptide mixture was treated (lower panel) or not (upper panel) with 5 mM DTT. (**D**) MALDI-TOF mass spectrum of DTT-treated HMGB1-ΔC after internal calibration on mono- and di-charged ions of apomyoglobin. The experimental masses of 23,365.3 Da and 23,232.7 Da correspond to the theoretical masses calculated for the recombinant HMGB1-ΔC containing (23,367.9 Da) or not (23,236.7 Da) the N-terminal methionine, respectively. Minor peaks with an asterisk correspond to either matrix adducts or N-terminal gluconoylation of the His-tagged protein. (**E**) HeLa cells were treated for 5 days with increasing concentrations of HMGB1-∆C (range: 5–200 nM) in the presence (open square) or the absence (black square) of 5 mM DTT. Prior to treatment, HMGB1-∆C was incubated for 2 h at 4 °C with 5 mM of DTT. Cell viability was assessed by the MTT assay. DTT alone was used as a control. Standard deviations (SDs) are indicated by error bars when they exceed symbol size.

HMGB1-ΔC is a truncated form of HMGB1 corresponding to its N-terminal part ([1–185] amino acid sequence). It was expressed as a fusion protein with a poly-histidine tag from pET22b(pelB-)HMGB1-ΔC (Figure 1A). Purification of either the truncated or the full-length protein was performed as described by Ni-NTA affinity chromatography [38,39]. To determine the redox status of the purified protein, HMGB1-ΔC was first subjected to SDS-PAGE under non-reducing or reducing conditions (Figure 1B). In the absence of β-mercaptoethanol, two bands corresponding to monomeric HMGB1-ΔC were observed. However, after treatment with β-mercaptoethanol, only the upper band (i.e., the reduced form) was still present, suggesting that the recombinant protein could exist in two different redox states. To confirm that an internal disulfide bond exists within HMGB1-ΔC, peptide mass fingerprinting experiments were conducted (Figure 1C). After in-gel digestion, extracted peptides were analyzed by matrix-assisted laser desorption/ionization–time of flight mass spectrometry (MALDI-TOF MS). In the mass spectrum acquired in the absence of DTT, we identified three peaks harboring m/z values that correspond to [M13-R24], [M13-K28] and [M13-K29] peptides linked by a disulfide bond with the [C45-R48] peptide (Figure 1C, upper panel). Consistently, these peaks totally disappeared in the presence of DTT and three other peaks appeared, corresponding to the [M13-R24], [M13-K28] and [M13-K29] peptides with their internal cysteine 23 harboring a free thiol (Figure 1C, lower panel). Altogether, these results suggest that both cysteines 23 and 45 are involved in the formation of an intra-molecular disulfide bridge that is present in the purified HMGB1-ΔC protein. Furthermore, MALDI-TOF MS analysis of intact HMGB1-ΔC protein after DTT treatment revealed the presence of two HMGB1-ΔC isoforms in which the N-terminal methionine was either excised or not (Figure 1D). To determine whether the redox state of HMGB1-ΔC might affect its hypothetical cytotoxic activity towards cancer cells, HeLa cervix carcinoma cells were treated with HMGB1-∆C in the presence or absence of DTT and the cell viability assessed (Figure 1E). No difference was detected, suggesting that both reduced and oxidized forms of HMGB1-∆C equally act on cancer cells. To confirm that the redox status of the truncated form of HMGB1 does not impact its anticancer activity in vitro, a triple mutant form C23-45-106S of HMGB1-ΔC (HMGB1-ΔC-TM) was constructed, purified and its activity tested (Supplementary Materials and methods and Figure S1). As expected, in the absence of β-mercaptoethanol treatment, only one band corresponding to monomeric HMGB1-ΔC-TM was detected (Figure S1B). Importantly, no differences could be detected between HMGB1-ΔC and HMGB1-ΔC-TM on the viability of HeLa cells (Figure S1C) as well as of HT-29 and HCT-116 cells (Figure S1D). These results confirm that HMGB1-∆C cytotoxic activity was independent of its redox state.

### 2.2. Comparison of the In Vitro Anticancer Activities of HMGB1-ΔC and HMGB1-fl on a Panel of Cancer Cell Lines in 2D/3D Cell Cultures

**Figure 2:**
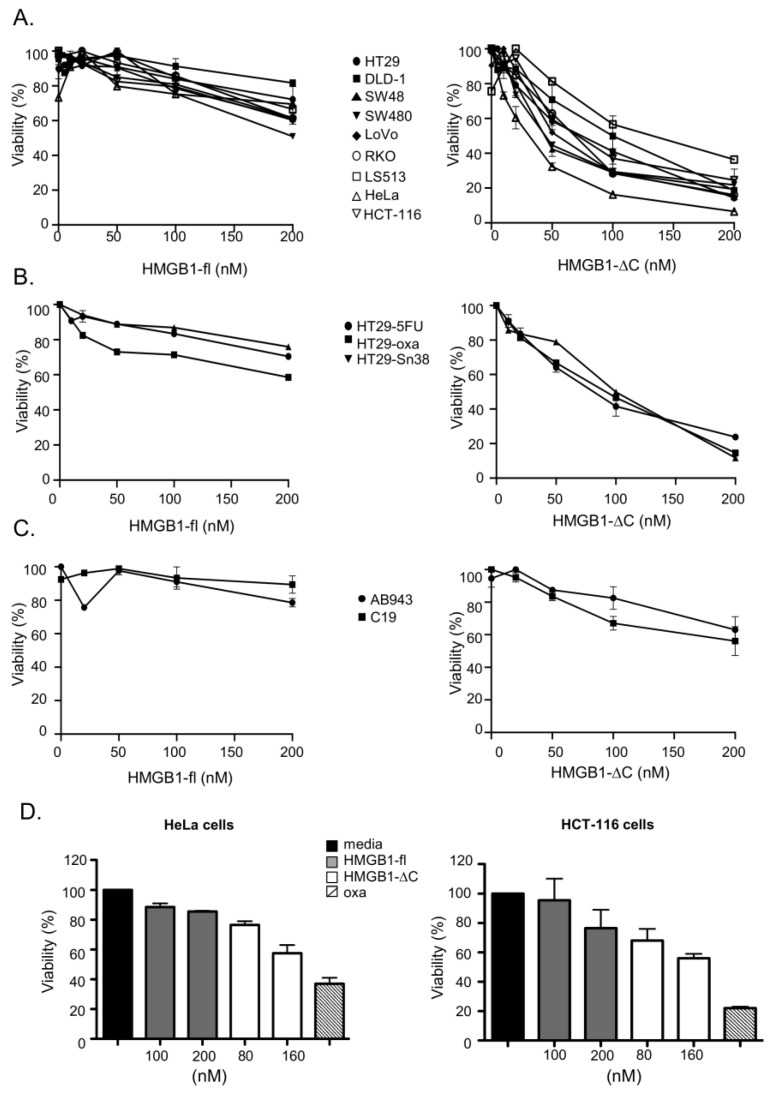
Cell viability assay of HMGB1-fl and HMGB1-∆C on human cancer and fibroblastic cell lines. (**A**–**C**) Two-dimensional cell viability assay: (**A**) CRC (HT-29, DLD-1, SW48, SW480, LoVo, RKO, LS513) and cervix carcinoma (HeLa) cells were treated for 5 days with increasing concentrations of HMGB1-fl or HMGB1-∆C (range: 5–200 nM) and cell viability assessed by the MTT assay; (**B**) HT-29 cells resistant to 5-fluorouracil (HT-29-5-FU), oxaliplatin (HT29-Oxa) and irinotecan (HT-29-Sn-38) were treated for 5 days with increasing concentrations of HMGB1-fl or HMGB1-∆C (range: 5–200 nM) and cell viability assessed by the MTT assay; (**C**) immortalized fibroblastic cells (C19 and AB943) were treated for 5 days with increasing concentrations of HMGB1-fl or HMGB1-∆C (range 5–200 nM) and cell viability assessed by the MTT assay. (**D**) Three-dimensional cell viability assay: HeLa and HCT-116 carcinoma cells were cultured in 3D and then treated with increasing concentrations of HMGB1-fl (100 nM, 200 nM) and HMGB1-∆C (80 nM, 160 nM) for 4 days. Cell viability was assessed by the CellTiter-Glo 3D assay. Oxaliplatin (Oxa, 4 µM) was used as a positive control. SDs are indicated by error bars when they exceed symbol size.

To determine whether the loss of the C-terminal tail impacts the previously reported anticancer activity of HMGB1-fl, the activities of the full-length and truncated forms of HMGB1 were evaluated on the HeLa cervix carcinoma cell line and a panel of colorectal cancer (CRC) cells with different genetic and phenotypic profiles (Table S1). Our results demonstrate that HMGB1-ΔC is consistently more active on cancer cells than its full-length counterpart, suggesting that the acidic tail might negatively modulate HMGB1 cytotoxic activity (Figure 2A). Importantly, our data show that colorectal cancer cells are sensitive to HMGB1 independently of their genetic (MSI/MSS, *BRAF* WT/*BRAF* mutated, *KRAS* WT/*KRAS* mutated and *TP53* WT/*TP53* mutated) and phenotypic (epithelial/mesenchymal) status. Moreover, cells that developed resistance to the DNA targeting agents associated with an increased stemness/mesenchymal phenotype (5-FU, oxaliplatin and SN-38) [40] still remain sensitive to HMGB1 with a more pronounced effect of the truncated form (Figure 2B). Strikingly, all cancer cells showed an IC50 value below those determined for two human immortalized fibroblastic cell lines (AB943 and C19) (Figure 2C). This suggests that HMGB1, and more specifically HMGB1-ΔC, preferentially targets tumor cells. Finally, because 2D cell cultures have limitations that might impact the response to anticancer agents, we evaluated the capability of both HMGB1-fl and HMGB1-ΔC to inhibit the growth of 3D tumor cell spheroids which are believed to more closely mimic in vivo conditions (Figure 2D). Again, HMGB1-ΔC showed a more pronounced effect than HMGB1-fl on the two cell lines tested (HeLa and HCT-116).

### 2.3. Cytotoxic Versus Cytostatic Activities of HMGB1-fl and HMGB1-ΔC

**Figure 3:**
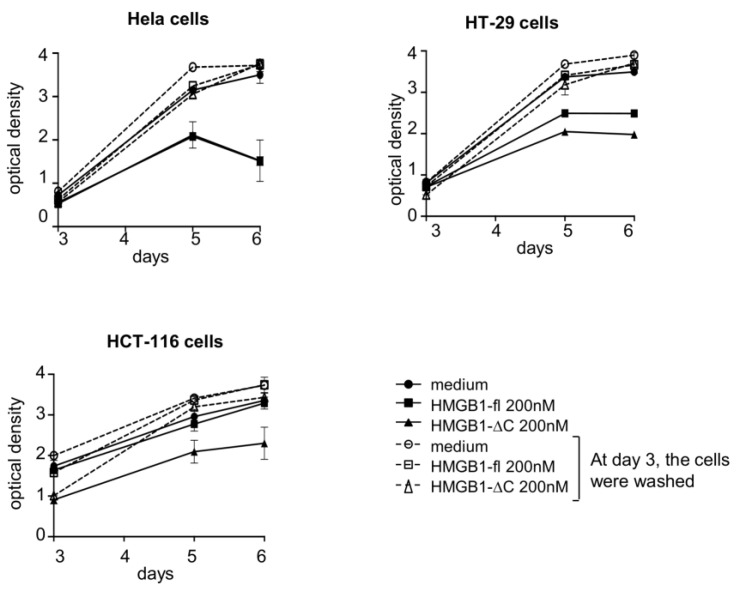
Cytotoxic versus cytostatic activities of HMGB1-fl and HMGB1-∆C on cancer cells. HeLa, HT-29 and HCT-116 carcinoma cells were treated for 6 days (black symbol) with a fixed concentration (200 nM) of HMGB1-fl or HMGB1-∆C. Alternatively, cells were treated for 3 days with a fixed concentration (200 nM) of HMGB1-fl or HMGB1-∆C, washed and then grown for 3 additional days in a fresh HMGB1-free medium (open symbol). At days 3, 5 and 6, cells were stained by crystal violet and cell survival was assessed by measuring the optical density at 570 nm. SDs are indicated by error bars when they exceed symbol size. On the HeLa cell panel, the two solid lines corresponding to the 6-day treatment with either HMGB1-fl (black square) or HMGB1-∆C (black triangle) appeared as merged on the graph.

Because cell viability assays do not allow to strictly distinguish between a cytotoxic effect and a cytostatic one, we evaluated the capability of both forms of HMGB1 to inhibit cell proliferation (Figure 3, solid lines). As previously reported for U251MG glioblastoma cells, a 6-day exposure period to HMGB1-fl resulted in a strong inhibition of proliferation of the HeLa, HT-29 and HCT-116 cell lines [17]. This result was confirmed for HMGB1-ΔC, which exhibited either a more pronounced effect (for HT-29 and HCT-116 cells) or a similar one (for HeLa cells) to that of the full-length protein. Surprisingly, a decrease in the number of tumor cells could only be observed for the HeLa cell line when treated with 200 nM of either HMGB1-fl or HMGB1-ΔC. On the contrary, exposure of both HCT-116 and HT-29 cell lines to the same concentration of HMGB1-fl and HMGB1-ΔC only led to cell growth arrest. These results demonstrate that HMGB1 induced various responses on tumor cells. Interestingly, when cells were washed after 3 days of treatment with HMGB1-fl or HMGB1-ΔC and then cultured for 3 additional days in a HMGB1-free medium, the effect of the two proteins on the tumor cells growth was abolished (Figure 3, dashed lines), suggesting that both full-length and truncated forms of HMGB1 required a continuous exposure time to optimally exert their anti-proliferative activities.

**Figure 4:**
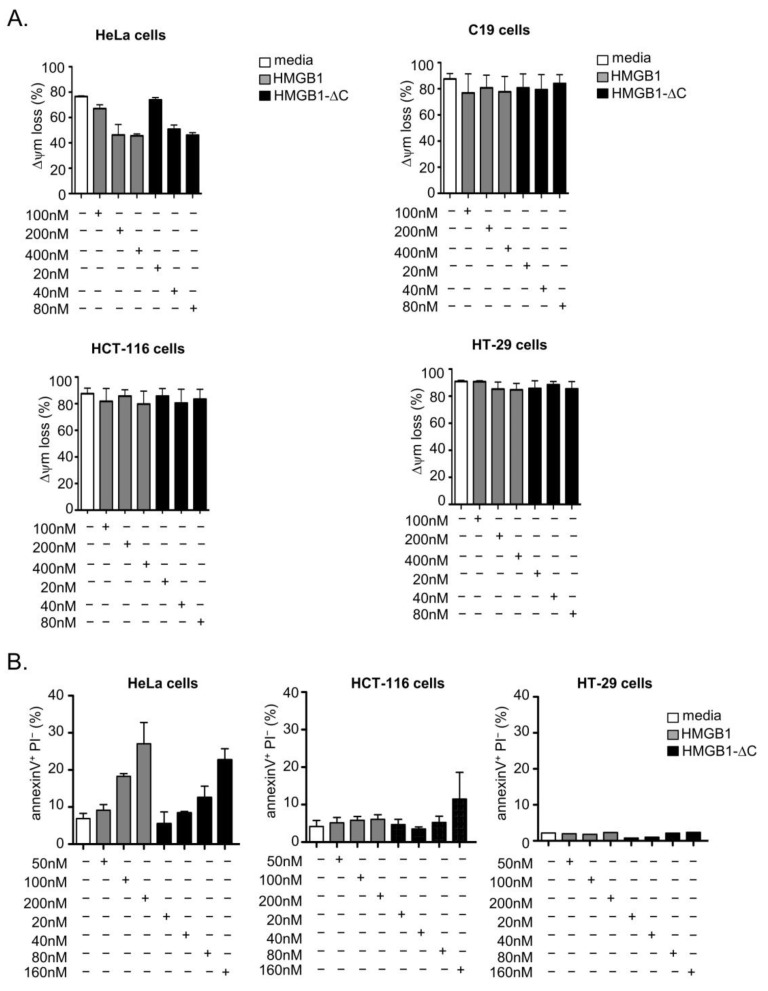
Mitochondrial membrane potential loss and apoptosis induction in cells treated with HMGB1-fl or HMGB1-∆C. (**A**) The HeLa, HCT-116 and HT-29 carcinoma cells as well as the C19 fibroblastic cell line were treated for 48 h with increasing concentrations of HMGB1-fl (range: 100–400 nM) or HMGB1-∆C (range: 20–80 nM). ∆ψm was determined by labeling cells with DIOC6(3) and fluorescence intensities quantified by flow cytometry. (**B**) The HeLa, HCT-116 and HT-29 cells were treated for 96 h with increasing concentrations of HMGB1-fl (range: 50–200 nM) or HMGB1-∆C (range: 20–160 nM) and labeled with Annexin V/PI. The percentage of early apoptotic cells (Annexin V^+^/PI^−^) was determined by flow cytometry. Data are expressed as mean ± SD. SDs are indicated by error bars when they exceed symbol size.

To confirm the dual effect of HMGB1-fl and HMGB1-ΔC on tumor cells, we first measured the influence of both forms of HMGB1 on the mitochondrial membrane potential (Δψm). This parameter is indeed considered as a key indicator of cell health or injury [41]. Moreover, mitochondria have been reported as major targets for HMGB1 anticancer activity [17]. To allow an accurate monitoring of Δψm rather than the plasmatic membrane potential (Δψp), we used here the DiOC6(3) fluorescent dye, known to accumulate in functional mitochondria [41]. Interestingly, at isotoxic doses, both HMGB1-fl and HMGB1-ΔC decreased, by 48 h of treatment, the DiOC6(3) staining intensity in HeLa cells (Figure 4A upper left panel and Figure S2), suggesting that both forms of HMGB1 are able to decrease the mitochondrial membrane potential in these cells. However, in the same conditions, neither HMGB1-fl nor HMGB1-ΔC could modulate Δψm in normal fibroblastic C19 cells (Figure 4A upper right panel and Figure S2) or in the two colon carcinoma cell lines HT-29 and HCT-116 (Figure 4A lower panels). To determine whether the changes in Δψm were associated with a subsequent induction of apoptosis, HeLa, HT-29 and HCT-116 cells were treated for 96 h with isotoxic concentrations of either HMGB1-fl or HMGB1-∆C and the percentage of apoptotic cells was determined by Annexin V/propidium iodide (PI) labeling (Figure 4B). Again, only the HeLa cells demonstrated a dose-dependent increase of early apoptotic labelling (Annexin V^+^/PI^−^). The absence of non-viable HCT-116 and HT-29 cells was further confirmed by single propidium iodide labeling done after 96 h of treatment with either HMGB1-fl or HMGB1-∆C (Figure S3). Together, our data show that both forms of HMGB1 can act on tumor cell fate by inducing either a cytotoxic or a cytostatic effect.

### 2.4. In Silico HMGB1-ΔC–PKM2 Docking

**Figure 5:**
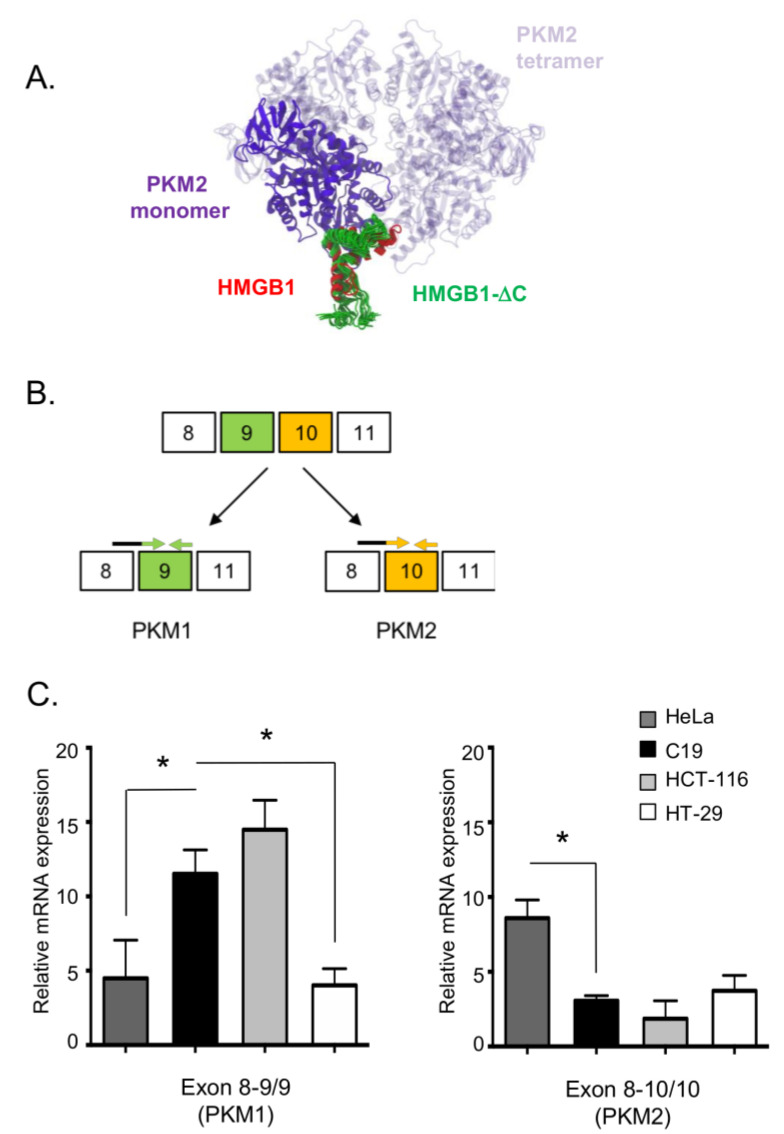
(**A**) In silico HMGB1-ΔC–PKM2 docking. Complexes were obtained after 40,000 simulations of diffusional association (SDA). The most favorable solutions were obtained after clustering and energy calculations. The PKM2 monomer considered in our calculations is colored purple while the other subunits are represented in high transparency. The lowest energy docking solution obtained for native HMGB1 is represented in red and the solutions obtained for HMGB1-ΔC are shown in green. (**B**) Schematic PKM-spliced isoforms. *PKM* gene encodes for two isoforms, PKM1 and PKM2. PKM1 only contains exon 9 (exon 10 is excised), whereas PKM2 only contains exon 10 (exon 9 is excised). Forward primers overlapping the exons 8–9 (PKM1) or the exons 8–10 (PKM2) junctions are shown in black/green and black/orange, respectively. Reverse primers specific to exon 9 or exon 10 are shown in green and orange, respectively. (**C**) Splicing pattern of *PKM* gene in HeLa, C19, HCT-116 and HT-29 cells. Transcript expression levels were measured by real-time PCR relative and fold changes calculation performed by using the 2^−ΔΔCT^ method. The mRNA expression levels of the housekeeping gene *L19* and of the constitutive exon2 of PKM1/2 were used for normalization. Data are expressed as mean ± SD. *p* values were calculated using the Student’s paired *t* test. * *p* < 0.05.

HMGB1 has been reported as an allosteric inhibitor of the active tetrameric form of PKM2, leading to the inhibition of the glucose–driven aerobic respiration [18]. To verify whether the modifications introduced in HMGB1-ΔC could hinder the interaction with PKM2, we performed docking calculations as previously described by Gdynia and collaborators [18] (Figure 5A). In our model, 78% of the complexes presenting the most favorable interaction energies were located at the binding site that was previously reported for the full-length protein. The partial disruption of electrostatic complementarity due to the structural modifications introduced in HMGB1 is likely to underlie the decrease in the rate of specific binding we observed. However, absolute measurements of binding affinities between the obtained complexes are beyond the limits of resolution of the method employed. Therefore, our results suggest that, in relative terms to those previously reported, HMGB1-ΔC retains the ability to interact with PKM2 at the binding site occupied by the native form. Because the poly-phosphorylated status of the HMGB1 B box was proposed to be an important factor for the specific binding of HMGB1 to PKM2 [18], we wondered whether the purified HMGB1-ΔC protein used herein was phosphorylated. MALDI-TOF MS analysis of the intact protein revealed that purified HMGB1-ΔC was not phosphorylated since no additional peak exhibiting a +98 Da mass increase was observed compared to the HMGB1-ΔC expected peaks (Figure 1D).

### 2.5. Expression Levels of PKM1/PKM2 mRNA in C19, HeLa, HT-29 and HCT-116 Cells

To determine whether the different cellular responses to HMGB1 and HMGB1-ΔC treatments might be linked to differential expression levels of PKM1/PKM2 isoforms, we measured transcript expression of the two isoforms by real-time PCR. In order to distinguish between PKM1 and PKM2 transcripts, forward primers overlapping the exons 8–9 (PKM1) or the exons 8–10 (PKM2) junctions were chosen and combined with reverse primers specific to exon 9 or exon 10, respectively (Figure 5B). To compare the expression levels of both transcripts and to detect alternative splicing events, an exon-level expression analysis was performed in which the amplification of the constitutive exon 2 was used to measure the global *PKM* gene expression level. As expected, the C19 fibroblastic cell line expressed higher levels of the PKM1 isoform compared to its PKM2 counterpart (Figure 5C). On the contrary, the HeLa cell line expressed higher levels of PKM2 in comparison to PKM1. Importantly, the differences observed at the transcriptional level were confirmed by immunoblotting (Supplementary Materials and methods and Figure S4). Noticeably, a marked difference in the level of expression of the PKM1 protein could be observed between the two cell lines and distinguished the C19 from the HeLa cells (Figure S4). In contrast to the profiles seen for the C19 and HeLa cell lines, the transcriptional levels of the two PKM isoforms in the HCT-116 and HT-29 cells were more unanticipated. On the first hand, the expression profile of the HCT-116 cells indeed resembles the profile that was expected for non-transformed cells with a high expression level of PKM1 and a low expression of PKM2. On the second hand, the HT-29 cells express low levels of both transcripts (Figure 5C). Therefore, whereas our data confirmed that non-transformed cells mostly express the PKM1 isoform, we could not strictly associate specific patterns for PKM1 and PKM2 relative expression in tumor cells.

### 2.6. Metabolic Effects of HMGB1-fl and HMGB1-ΔC on C19, HeLa, HT-29 and HCT-116 Cells

**Figure 6:**
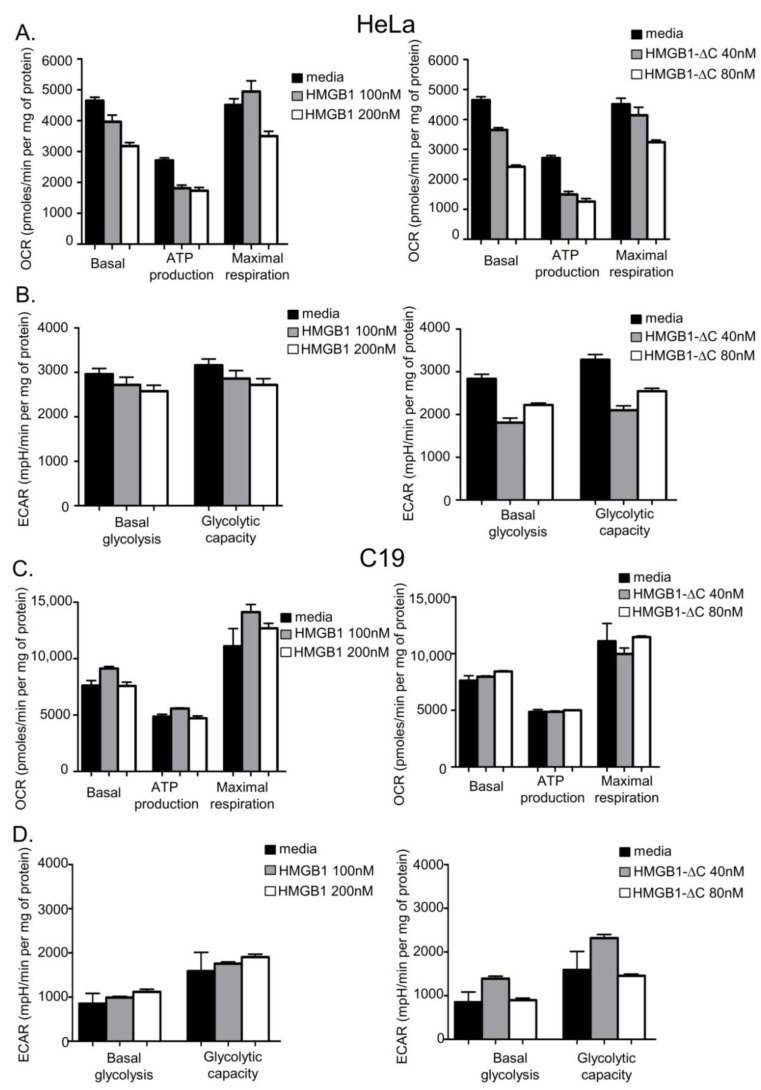
Acute effects of increasing concentrations of HMGB1-fl and HMGB1-∆C on HeLa (**A**,**B**) and C19 (**C**,**D**) cell bioenergetics. (**A**) HeLa cells were seeded in a Seahorse XFe24 plate, treated for 48 h with HMGB1-fl (left panel) or HMGB1-∆C (right panel) and basal OCR measurements were made followed by injection of oligomycin (1 µM), FCCP (1 µM) and rotenone (10 µM) + antimycin A (10 µM). ATP linked respiration is the difference in OCR before and after oligomycin injection. Maximal respiration is defined as the OCR after FCCP injection. Non-mitochondrial respiration was determined after rotenone + antimycin A injection. The rates of basal respiration, ATP-linked respiration and maximal respiratory capacity were normalized with the total amount of proteins present in each well. OCR is expressed in pmoles per min per mg of protein. The data shown are representative of 3 independent experiments. Data are expressed as mean ± SD. (**B**) Simultaneously to OCR measurements, ECAR was quantified on HMGB1-fl (left panel) or HMGB1-∆C (right panel) treated HeLa cells before (basal glycolysis) and after (maximal glycolytic capacity) oligomycin injection. Basal glycolysis and the maximal glycolytic capacity were normalized with the total amount of proteins present in each well. ECAR is expressed in mpH per min per mg of protein. The data shown are representative of 3 independent experiments. Data are expressed as mean ± SD. (**C**) Same as (**A**), except that C19 cells basal respiration, ATP-linked respiration and maximal respiratory capacity were determined after treatment with HMGB1-fl (left panel) or HMGB1-∆C (right panel). (**D**) Same as (**B**), except that ECAR was quantified on HMGB1-fl (left panel) or HMGB1-∆C (right panel) treated C19 cells before (basal glycolysis) and after (maximal glycolytic capacity) oligomycin injection.

**Figure 7:**
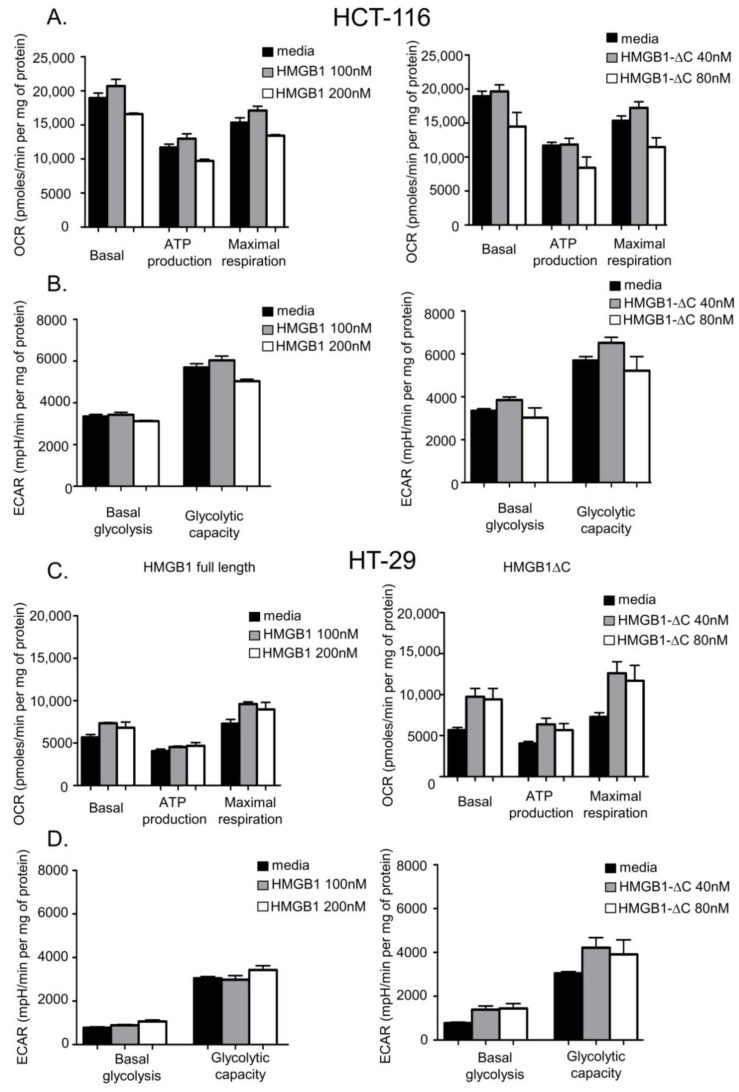
Acute effects of increasing concentrations of HMGB1-fl and HMGB1-∆C on HCT-116 (**A**,**B**) and HT-29 (**C**,**D**) cell bioenergetics. (**A**) HCT-116 cells were seeded in a Seahorse XFe24 plate, treated for 48 h with HMGB1-fl (left panel) or HMGB1-∆C (right panel) and basal OCR measurements were made followed by injection of oligomycin (1 µM), FCCP (1 µM) and rotenone (10 µM) + antimycin A (10 µM). ATP linked respiration is the difference in OCR before and after oligomycin injection. Maximal respiration is defined as the OCR after FCCP injection. Non mitochondrial respiration was determined after rotenone + antimycin A injection. The rates of basal respiration, ATP-linked respiration and maximal respiratory capacity were normalized with the total amount of proteins present in each well. OCR is expressed in pmoles per min per mg of protein. The data shown are representative of 3 independent experiments. Data are expressed as mean ± SD. (**B**) Simultaneously to OCR measurements, ECAR was quantified on HMGB1-fl (left panel) or HMGB1-∆C (right panel) treated HCT-116 cells before (basal glycolysis) and after (maximal glycolytic capacity) oligomycin injection. Basal glycolysis and the maximal glycolytic capacity were normalized with the total amount of proteins present in each well. ECAR is expressed in mpH per min per mg of protein. The data shown are representative of 3 independent experiments. Data are expressed as mean ± SD. (**C**) Same as (**A**), except that HT-29 cells basal respiration, ATP-linked respiration and maximal respiratory capacity were determined after treatment with HMGB1-fl (left panel) or HMGB1-∆C (right panel). (**D**) Same as (**B**), except that ECAR was quantified on HMGB1-fl (left panel) or HMGB1-∆C (right panel) treated HT-29 cells before (basal glycolysis) and after (maximal glycolytic capacity) oligomycin injection.

To assess whether the loss of the C-terminal region of HMGB1 might impact the ability of the full-length protein to impair aerobic respiration in cancer cells, we tested the effects of both forms of HMGB1 on cellular bioenergetics. The Seahorse technology was used because it allows simultaneous measurements of the basal oxygen consumption rate (OCR) and extracellular acidification rate (ECAR), two parameters that characterize basal respiration and glycolysis, respectively. Interestingly, HeLa cells treatment with isotoxic concentrations of either HMGB1-fl or HMGB1-ΔC led to a dose-dependent decrease of their basal respiration capacity (Figure 6A and Figure S5A). Moreover, HMGB1-ΔC, but not HMGB1-fl, strongly diminished the basal glycolytic capacity of the cells (Figure 6B and Figure S5B). In contrast, both forms of HMGB1 did not noticeably affect the basal respiration and glycolytic capacities of the C19 fibroblastic cells (Figure 6C,D and Figure S6). Similarly, HMGB1-fl or HMGB1-ΔC did not impact glycolysis in the HCT-116 cell line while basal respiration was slightly decreased at 200 nM and 80 nM of HMGB1-fl and HMGB1-ΔC, respectively (Figure 7A,B and Figure S7). However, the impact of this decrease on the bioenergetics in these cells is likely to be negligible if we consider the high basal respiration level reached in HCT-116 cells compared to the three other cell lines. Intriguingly, the HT-29 cell line showed a specific pattern in which basal respiration was increased after treatment with both forms of HMGB1 (Figure 7C and Figure S8A). A more pronounced effect was, however, observed for the truncated form (Figure 7C, right panel). In addition, HMGB1-ΔC, but not HMGB1-fl, increased the basal glycolytic capacity of these cells, suggesting that the truncated protein might stimulate cellular bioenergetics instead of inhibiting it in HT-29 cells (Figure 7D and Figure S8B).

To further characterize the action of both forms of HMGB1 on cellular bioenergetics, oligomycin, an inhibitor of the ATP synthase (complex V), was added to our samples. The addition of oligomycin indeed helps to evaluate the OCR value that is directly linked to ATP production. For the four cell lines tested, the effects of both HMGB1-fl and HMGB1-ΔC that were observed for basal respiration were mostly recapitulated on the portion of basal respiration that is effectively used to drive ATP production (compare basal respiration and ATP production histograms on Figure 6A,C and Figure 7A,C). These results indicate that HMGB1 primarily acts on respiration that is involved in ATP production. In addition to its effect on OCR, oligomycin treatment also permits the measurement of the maximum ECAR that is usually reached when oxidative phosphorylation arrest drives the cell to use glycolysis to its maximum capacity. Interestingly, while glycolysis is increased in the C19, HCT-116 and HT-29 cells following treatment, the addition of oligomycin to HeLa cells did not modify the ECAR values, suggesting that glycolysis is already working at its maximum capacity in these cells (compare basal glycolysis and glycolytic capacity histograms in Figure 6B,D and Figure 7B,D). In all cases, however, the effects of both forms of HMGB1 on the maximum glycolytic capacity were very similar to what was found for basal glycolysis (compare basal glycolysis and glycolytic capacity histograms in Figure 6B,D and Figure 7B,D).

Finally, to determine whether HMGB1-fl and HMGB1-ΔC treatments act on the mitochondrial electron transport chain (ETC) independently of ATP production, the uncoupler carbonyl cyanide 4-(trifluoromethoxy)phenylhydrazone (FCCP) was added to our samples. After treatment, the maximal respiration capacity was evaluated by calculating the difference between maximal and basal OCR (Figure 6A,C, Figure 7A,C and Figures S5A, S6A, S7A and S8A). Again, the effects of both HMGB1-fl and HMGB1-ΔC on the maximal respiration capacity of the four cell lines mostly recapitulated what was observed for basal respiration (compare basal respiration and maximal respiration histograms on Figure 6A,B, and Figure 7A,C). These data suggest that HMGB1-fl and HMGB1-ΔC act on cell bioenergetics either by directly affecting ETC or by modulating its upstream substrates.

## 3. Discussion

The C-terminal acidic tail of HMGB1 ([186–215] amino acid sequence) is known to modulate its biological functions. However, its capacity to act on its anticancer activity in vitro has not been studied yet. In this study, we show that the deletion of the C-terminal acidic tail of HMGB1 increases its antitumor activity towards a large panel of cancer cells, including cells that are otherwise resistant to DNA-targeting agents. Interestingly, while the C-terminal acidic tail of HMGB1 has been reported to play a key role in its antibacterial activity [37], our results demonstrate that the same region negatively modulates its anticancer properties. One might suggest that the propensity of the acidic tail of HMGB1 to bind to the HMG boxes in vitro could interfere with its capacity to bind PKM2, a recently identified target of HMGB1 [18]. However, our docking calculations using the same protocol as Gdynia and collaborators demonstrated that HMGB1-ΔC retains the ability to interact with PKM2 at the same binding site as HMGB1-fl with a similar or even lower rate of specific binding. Therefore, our results suggest that the deletion of the C-terminal acidic tail of HMGB1 is unlikely to increase its ability to interact with and inhibit the active tetrameric form of PKM2. Further studies will have to address this specific question.

Another interesting point raised by our study is how HMGB1 exerts its anticancer activity in terms of cell viability. Indeed, it was initially proposed that HMGB1 induces a novel form of cell death [17]. In glioma cells, this distinct form of necrotic cell death was characterized by the formation of giant mitochondria followed by the loss of plasma membrane integrity measured through lactate dehydrogenase release. However, in colorectal cancer cells, while the formation of giant mitochondria was also described, cell death induction was not strictly demonstrated since only cell viability assays were performed [17,18]. In that context, both crystal violet staining and MTT assay, which are often used to assess cell viability of proliferating cells, do not allow to discriminate between anti-proliferative effect, cell death induction, cell cycle or metabolism alteration [42,43]. In our hands, by combining viability (crystal violet staining and MTT assay) and cytotoxicity (Δψm loss and Annexin/PI labeling) assays, we were not able to detect any form of cell death induction for both HCT-116 and HT-29 cells following HMGB1-fl or HMGB1-ΔC treatments while their “viability” was affected. This suggests that both forms of HMGB1 might exert a cytostatic effect on HCT-116 and HT-29. In contrast, HeLa cells univocally showed cell death induction through apoptosis. Together, these data led us to assume that HMGB1 might exert its in vitro anticancer activity through a new form of necrotic cell death, apoptosis or cytostasis. Importantly, these effects seem to be limited to cancer cells since none of the HMGB1 forms impacted the cellular viability or the metabolic activity of the C19 fibroblastic cell line. This is in agreement with previous data obtained by others on astrocytes [17]. Interestingly, the outcome of HMGB1 treatment might be quantitatively and qualitatively linked to PKM1/2 expression as the latter isoform is expressed in many types of cancer cells but not in their normal cellular counterparts [7,8,9,10,11,12,13,14]. In that sense, contrary to C19 cells, HeLa cells which predominantly express PKM2 are highly sensitive towards HMGB1-ΔC both in terms of metabolic (e.g., aerobic respiration and glycolysis) and cytotoxic activities. However, PKM2 expression levels cannot be used as a unique biomarker to predict cancer cell response towards HMGB1 derivatives. This is particularly true for cells in which other metabolic pathways such as glutaminolysis are used for mitochondrial ATP generation [18]. Accordingly, we show here that treatment of HT-29 cells by HMGB1-ΔC is accompanied by an increased respiratory activity, suggesting that inhibition of PKM2 might stimulate alternative metabolic pathways. Moreover, since PKM2 activity is known to be modulated by various allosteric regulations in response to upstream glycolytic or purine nucleotide synthesis intermediates, acting on cancer cell metabolism might also affect the equilibrium between the high- and low-activity states of PKM2, making response prediction even more uncertain [13]. Thus, a major challenge consists in characterizing the state of PKM2 that predominates in the cells. This is particularly true since small molecules have recently been developed to selectively activate PKM2 by enhancing its association into stable tetramers, meaning that targeting both states of PKM2 results in anticancer properties [9]. Interestingly, these limitations might be circumvented through the identification of biomarkers that are associated with the non-metabolic functions of dimeric PKM2. For example, the phosphorylation of dimeric PKM2 by serine/threonine kinases or its acetylation on lysine 433 have been reported to favor its nuclear translocation and transcriptional activities [44,45,46,47]. Similarly, it has been reported that the direct phosphorylation of PKM2 on tyrosine 105 inhibits the formation of active tetramers by disrupting binding of fructose-1,6-bisphosphate to PKM2 [48]. Thus, assessing PKM2 post-translational modifications in cells might help to predict tumor cell responses towards PKM2 activators or inhibitors and thus orientate therapeutic decisions.

Finally, Gdynia and co-workers suggested that HMGB1-fl interacts with PKM2 when HMGB1 is phosphorylated on its tyrosine residues [18]. In terms of therapeutics production, this might be a major drawback to ensure efficient bioprocessing and scale-up as well as the quality and reproducibility of therapeutic forms of HMGB1. Moreover, high scale production of therapeutic proteins in mammalian cells might induce elevated costs [49]. However, we and others have shown that recombinant HMGB1 expressed and purified from bacterial sources maintains similar in vitro anticancer properties to HMGB1 purified from mammalian cells. To explain this phenomenon, it was suggested that bacterial endokinases might poly-phosphorylate recombinant HMGB1 [18]. However, in the present study, no phosphorylated residue was detected by mass spectrometry, suggesting that both the full-length and the mutant recombinant forms of HMGB1 were not phosphorylated. Therefore, HMGB1 may possibly be phosphorylated following internalization. Another limitation of using HMGB1 as an anticancer agent in patients might come from the existence of various oxidized and reduced forms of HMGB1 [20,22,25]. In particular, systemic administration of recombinant HMGB1 might not predict in which redox state the proteins will be in the vicinity of the tumor. The tumor microenvironment might indeed alter the redox status of the three conserved cysteines which are sensitive to oxidation and thus modify the anticancer activities of HMGB1-fl and its derivatives. In that context, we here show that oxidized forms of HMGB1-∆C retain their in vitro anticancer activity when treated with DTT. Moreover, the replacement of cysteines 23, 45 and 106 by serine residues in HMGB1-ΔC-TM did not impact the anti-proliferative activity of HMGB1-ΔC, thus suggesting that the redox status of the recombinant proteins is not a key determinant for their direct activity on tumor cells. However, predicting side effects in vivo remains difficult. Others have shown that HMGB1-fl can be safely administered to athymic nude mice [17,18]. Nonetheless, in immune competent animals and in humans, altered redox states might have unpredictable consequences in terms of extracellular cytokine and proinflammatory functions of HMGB1 [20]. Further studies using immunocompetent and immunodeficient mice injected with syngeneic tumor cell lines will therefore be required to assess whether the in vitro anticancer activities of HMGB1 might be modulated in vivo by the immune system. In that context, HMGB1-ΔC-TM might serve as an efficient tool to circumvent the proinflammatory functions of disulfide-HMGB1. Indeed, it has been shown that the replacement of all the cysteines with serine in HMGB1-fl preserves or even improves the chemokine functions of the fully reduced form of the protein while it fully eliminates its cytokine-inducing activity. Moreover, this non-oxidizable mutant form of HMGB1-fl makes the protein resistant to inactivation by ROS [50]. Again, further studies will have to address these questions. In particular, it would be interesting to determine whether HMGB1-ΔC-TM might help combining cancer cell directed and immune therapies.

## 4. Materials and Methods

### 4.1. Cells

HeLa-M cervical carcinoma cells were obtained from ATCC (American Type Culture Collections, Manassas, VA, USA) while colorectal carcinoma cells were kindly provided by Richard Camalier (Division of Cancer Treatment and Diagnosis Tumor Repository, National Cancer Institute) and Richard Hamelin (Paris, France). 5-fluorouracil, SN-38 (7-Ethyl-10-hydroxycamptothecin) and oxaliplatin resistant HT-29 cell lines were a gift from Annette K. Larsen (Paris, France) [40,51]. C19 and AB943 fibroblasts were kindly provided by the Platform for Immortalization of Human Cells from the “Institut de Myologie” (Hôpital Pitié-Salpétrière, Paris, France). HeLa-M cervical carcinoma cells were maintained in RPMI with 10% fetal bovine serum (FBS), 100 units/mL penicillin and 100 µg/mL streptomycin (Gibco, Life Technologies, Carlsbad, CA, USA). Colon carcinoma cell lines and human immortalized fibroblasts were maintained in DMEM (except for HCT-116 cells that were cultured in McCoy medium) supplemented with 10% FBS and 100 units/mL penicillin and 100 µg/mL streptomycin.

### 4.2. Plasmid Construction

pET22b(pelB-)HMGB1-ΔC was constructed by cloning a truncated form of HMGB1 coding sequence in the pET22b(pelB-) expression vector. The DNA sequence corresponding to HMGB1 amino acid 1 to 185 was amplified by PCR using AmpliTaq gold master mix (Applied Biosystems) according to the manufacturer’s instructions and forward (5′TAAGAAGGAGATATACATATGGGCAAAGGAGATCCTAA3′) and reverse (5′GACGGAGCTCGAATTCGGCTTCTTTTTCTTGCTTTTTT3′) primers. The PCR product was then inserted in the pET22b(pelB-) expression vector at NdeI/EcoRI sites using an In-Fusion HD EcoDry cloning kit (Clontech). All constructs were validated through Sanger sequencing using the Big Dye Terminator v3.1 Cycle Sequencing Kit (Applied Biosystems, Waltham, MA, USA).

### 4.3. Protein Expression and Purification

Recombinant rat HMGB1 and HMGB1-ΔC were purified from *Escherichia coli* BL21(DE3) pLysS transformed with pET15b-6His-HMGB1 and pET-22b(pelB-)HMGB1-ΔC, respectively. Protein expression and purification were performed as previously described [38,39]. The removal of contaminating bacterial lipopolysaccharide was performed using Triton X-114, as described [38,52]. The eluted proteins were dialyzed against PBS and then submitted to 12% SDS-PAGE to determine the purity and stored in aliquots at −80 °C. The protein concentration was calculated using Bradford’s method.

### 4.4. In-Gel Digestion

Sixty-six microliters of recombinant HGBM1-ΔC protein (12.5 µg) was subjected to a cysteine-alkylation step in the presence of 4.5 mM of N-ethylmaleimide (Sigma-Aldrich, Saint Quentin-Fallavier, France) for 45 min at room temperature. Simultaneously, the sample was concentrated to 30 µL using a centrifugal vacuum concentrator (Eppendorf). Then, the sample was subjected to SDS-PAGE under non-reductive conditions using a 12% acrylamide gel. After Coomassie Blue staining, the band corresponding to the recombinant HGBM1-ΔC protein was excised manually, destained, dried by vacuum and digested as previously described [53]. Briefly, gel pieces were reswollen with 10 µL of proteomics grade Trypsin Gold (Promega, Charbonnières, France) prepared at 25 ng/µL in 1 mM HCl. Then, digestion was performed overnight at 37 °C by adding 100 µL of 50 mM ammonium bicarbonate (AMBIC, Sigma-Aldrich, Saint Quentin-Fallavier, France). The supernatant was kept and gel pieces were further extracted with 100 µL of 60% acetonitrile (ACN, Sigma-Aldrich, Saint Quentin-Fallavier, France). Supernatants were then pooled and concentrated to 100 µL using a centrifugal vacuum concentrator. Five microliters were treated or not with 5 mM DTT for 30 min at 37 °C and analyzed by MALDI-TOF mass spectrum.

### 4.5. MALDI-TOF Mass Spectrometry Analysis of HMGB1-ΔC

For peptide mass fingerprints, 1 µL of peptide mixtures was mixed with 2 µL of a half-saturated solution of α-cyano-4-hydroxycinnamic acid in 50/0.3 ACN/trifluoroacetic acid. Two microliters of this premix were spotted onto the sample plate and allowed to dry under a gentle air stream. Spectra were acquired in positive reflectron mode on an Axima Performance MALDI-TOF/TOF mass spectrometer (Shimadzu, Manchester, UK) with a pulse extraction fixed at 2500 as described in [54].

For intact mass MALDI-TOF MS analysis, 10 µL of recombinant HMGB1-ΔC was mixed with 1 µL of 250 mM AMBIC and 1 µL of 50 mM DTT freshly prepared in distilled water. After 1 h incubation at room temperature, 1 µL of prereduced HMGB1-ΔC was mixed with 1 µL of 1.5 µM of equine apomyoglobin solution and 1 µL of a saturated solution of sinapinic acid, both prepared in 30/0.3 ACN/trifluoroacetic acid. Then, 2 µL of this mixture was spotted on a MALDI sample plate and dried under a gentle air stream. MALDI-TOF MS analysis was performed as described in [55] except that mass spectrum was internally calibrated using mono- and di-charged ions of equine apomyoglobin as references.

### 4.6. 2D Cell Viability Assay

Cell viability was first determined by the methylthiazolyldiphenyl-tetrazolium bromide (MTT) assay as described previously [56]. Briefly, cells were exposed to the indicated concentrations of HMGB1-fl or HGBM1-ΔC for five doubling times and then incubated for 3 h at 37 °C with MTT. Formazan crystals were then dissolved in dimethylsulfoxide (DMSO) and optical density measured at 570 nm in a microplate reader (Tecan, Männedorf, Switzerland). The half-maximal inhibitory concentration (IC_50_) was determined. All values are averages of at least 3 independent experiments done in duplicate. Alternatively, cell growth and viability were assessed by crystal violet staining. Briefly, 3 × 10^3^ cells per well were incubated in 96-well plates and exposed to the indicated concentrations of HMGB1-fl or HGBM1-ΔC for 3, 5 or 6 days. Culture medium was then removed and the cells fixed for 15 min at room temperature with 4% paraformaldehyde. The cells were then stained for 5 min with a 0.05% crystal violet solution, washed three times with PBS and blue dyes dissolved in 200 μL of SDS 1%. Optical density was measured at 570 nm in a microplate reader (Tecan). All values are averages of at least 2 independent experiments done in duplicate.

### 4.7. 3D Cell Viability Assay

Cell viability in spheroids was determined by the CellTiter-Glo 3D luminescent cell viability assay (Promega) according to the manufacturer’s instructions. Briefly, 2000 cells were seeded in an ultra-low attachment 96-well round-bottom plate (Corning Inc., Corning, NY, USA). After 24 h, spheroids were treated with either HMGB1-fl (100 nM, 200 nM), HGBM1-ΔC (80 nM, 160 nM) or oxaliplatin (4 µM). After 4 days, spheroids were lysed and incubated at room temperature for 25 min. Luminescence was measured in a microplate reader (Tecan). All values are averages of at least 3 independent experiments.

### 4.8. Flow Cytometry

To evaluate the changes in the inner mitochondrial transmembrane potential (Δψm), the HeLa, C19, HCT-116 and HT-29 cells were treated for 48 h with HMGB1-fl (100, 200 and 400 nM) or HMGB1-∆C (20, 40 and 80 nM). Cells were then stained for 15 min at 37 °C with 40 nM of DiOC6(3) (Molecular Probes, Eugene, OR, USA) and analyzed on a FC500 flow cytometer (Beckman Coulter, Villepinte, France). After cell debris exclusion, DiOC6(3) fluorescent intensities were measured using the FlowJo or Kaluza software and the percentage of Δψm loss was calculated. All values are averages of 3 independent experiments.

To evaluate the induction of cell death, 2000 cells were seeded into a 96-well plate and treated with HMGB1-fl (50 nM, 100 nM, 200 nM) or HGBM1-ΔC (20 nM, 40 nM, 80 nM, 160 nM). After 4 days of incubation at 37 °C, cells were harvested and labeled with 0.1 mg/mL APC Annexin-V (BD Biosciences, Franklin Lakes, NJ, USA) and 0.5 mg/mL PI (Sigma, Saint Quentin-Fallavier, France). Fluorescent intensities were recorded using a Cytoflex Cytometer (Beckman Coulter, Villepinte, France) and the percentage of early apoptotic cells (PI negative, APC Annexin V positive) was calculated.

### 4.9. In Silico Protein Docking Studies

The simulation of diffusional association (SDA) method [57] was used as described previously [18]. Briefly, the monomeric PKM2 (PDB ID:3BJF) and HMGB1 box B (PDB ID:2YRQ, residues 95–163) were taken as fixed and mobile, respectively. To reproduce the results from Gdynia et al., tyrosine residues 116 and 162 (numbering according to PDB file) were phosphorylated using the build feature of UCSF Chimera [58]. To model the engineered HMGB1-ΔC, we used the Phyre2 web server [59]. The added residues were refined using the MODELLER loop refinement protocol [60] and the Discrete Optimized Protein Energy (DOPE) score was used for evaluating the quality of the models [61]. The model presenting the best score was selected for SDA calculations and its stereochemistry was validated using PROCHECK [62]. Prior to SDA calculations, all structures were prepared with the PDB2PQR program using the AMBER force field parameters, with protonation states assigned at pH 7 [63]. The phosphotyrosine parameters were taken from Homeyer et al. [64] and the Adaptive Poisson–Boltzmann Solver (APBS) version 1.4.1 [65] was used to solve the linearized Poisson–Boltzmann equation with a protein dielectric constant of 1 and a solute dielectric of 78 to calculate the electrostatic potential for each protein on cubic grids of 129 points, with 1 Å grid spacing. Extra effective charges were added on the phosphorus and oxygen atoms of the phosphotyrosine residue in addition to those placed by default during the SDA preparation steps. To perform the simulation of 40,000 encounter complexes between HMGB1 and PKM2, we applied the same simulation parameters and reaction criteria as those described by Gdynia et al. [18]. The 5000 docked complexes presenting the most favorable interaction energy were retained for analysis by the SDA clustering tool. The 10 most populated cluster solutions were retained for quantitative and visual analysis. All images were prepared using the VMD visualization software [66].

### 4.10. RNA Extraction and RT-qPCR Analysis

Total cellular RNA was isolated using TRIzol-reagent (Life Technologies) and subjected to DNase treatment (TURBO DNA-free kit; Life Technologies). Then, 2 µg of DNA-free RNA was reversed-transcribed to cDNA using the High-Capacity cDNA Reverse Transcription Kit (Thermo Scientific, Waltham, MA, USA). Transcript expression levels were measured by real-time PCR with the SensiFast SYBR No-Rox kit (Bioline, Toronto, ON, USA). Reactions were analyzed with a Biorad CFX96 Touch Real-Time PCR machine and relative gene expression levels were calculated in fold changes using the 2^−ΔΔCT^ method [67]. For normalizing mRNA expression levels, the L19 mRNA was used as a control. In addition to control gene normalization, constitutive PKM exon 2 normalization was performed for exon-level expression analysis. All values are averages of at least 3 independent experiments done in duplicate. Gene specific primers are listed in Table 1.

### 4.11. Measurement of Oxygen Consumption Rate (OCR) and Extracellular Acidification Rate (ECAR)

The mitochondrial OCR and ECAR were measured by using a Seahorse XFe24 analyzer (Agilent, Santa Clara, CA, USA) according to the manufacturer’s instructions. Briefly, 40,000 cells per well were seeded onto an XFe24 cell Culture Microplate (Seahorse Bioscience, Lexington, MA, USA) and incubated with either HMGB1-fl (at 50, 100 and 200 nM) or HGBM1-ΔC (at 10, 20 and 40 nM) for 48 h. Before measurements, the cellular growth medium was replaced with a serum-free RPMI (HeLa) or DMEM (C19, HT-29, HCT-116) medium (Agilent Seahorse XF RPMI Medium) complemented with 2 mM L-glutamine, 1 mM sodium pyruvate (pH 7.4) and glucose (2 g/L for HeLa; 4.5 g/L for C19, HT-29 and HCT-116). The cells were then incubated for 30 min at 37 °C in a CO_2_-free incubator. Basal OCR and ECAR were first measured over time in the absence of mitochondrial inhibitors. Then, 1 µM oligomycin (mitochondrial complex V inhibition), 1 µM FCCP (mitochondrial oxidative phosphorylation uncoupler) and a combination of 10 µM rotenone (Complex I inhibitor) and 10 µM antimycin A (Complex III inhibitor) were successively injected. The cellular bioenergetic parameters determined were ATP linked respiration, proton leak, maximal respiration and non-mitochondrial respiration (OCR) as well as maximal glycolytic capacity (ECAR). ATP linked respiration was derived from the difference between OCR at baseline and respiration following oligomycin addition. Proton leak corresponds to the remaining basal respiration not coupled to ATP production and is determined from the difference in OCR between oligomycin and ETC inhibitors (rotenone and antimycin A). Maximal respiration was determined by subtracting the OCR after the addition of rotenone and antimycin A from the OCR induced by FCCP. Non mitochondrial respiration was deduced from the OCR persisting after the addition of rotenone and antimycin A. At the end of the experiment, cells were lysed and the protein concentration calculated using Bradford’s method. All OCR and ECAR values were then normalized and expressed in pmol of O_2_/min/mg of protein and mph/min/mg of protein, respectively. All values are averages of three independent wells.

## 5. Conclusions

In summary, our study shows that the deletion of the C-terminal acidic tail of HMGB1 increases the in vitro anticancer activity of the recombinant protein towards a large panel of cancer cells without affecting normal immortalized fibroblasts. Moreover, our data demonstrate that the effect of the truncated protein is not dependent on its redox state, known to otherwise modulate the in vivo immunogenic activities of the protein. Finally, we show that HMGB1-ΔC retains the capacity of the full-length protein to perturb the metabolism of cancer cells even if the capacity of the cells to circumvent oxidative phosphorylation inhibition might determine their cellular outcome. Taken together, our study supports further works in order to characterize the in vivo anticancer activity of the recombinant wild-type and mutant forms of HMGB1 in immunocompetent animals.

## Figures and Tables

**Table 1:** Primers’ sequences.

Target	Forward (5′->3′)	Reverse (5′->3′)
Exon 8–9/9	ATGCAGCACCTGATAGCTCGTGA	TGCCAGACTCCGTCAGAACTATCA
Exon 8–10/10	ATGCAGCACCTGGTCTGCTCACC	CATTCATGGCAAAGTTCACCCGGA
Exon 2	CCATGTCGAAGCCCCATAGT	GCTGGGCCAATGGTACAGAT
*L19*	ATGTATCACAGCCTGTACCTG	CGTGCTTCCTTGGTCTTAGAC

## Data Availability

Not applicable.

## Supplementary Materials

The following are available online at https://www.mdpi.com/article/10.3390/ijms23147865/s1.
