# Supplementary material for: The C-Terminal Acidic Tail Modulates the Anticancer Properties of HMGB1
Source: Int J Mol Sci. 2022 Jul 17;23(14):7865. doi: 10.3390/ijms23147865 (PMC9319070; doi:10.3390/ijms23147865)
Supplement: Supplementary file 1 [file ijms-23-07865-s001.zip › ijms-1821737-supplementary.pdf]

SUPPLEMENTARY INFORMATIONS

Supplementary materials and methods.

HMGB1-ΔC-TM plasmid construction

The triple mutant form C23-45-106S of HMGB1-ΔC was constructed by directed mutagenesis using pET22b(pelB-) HMGB1-ΔC vector as a template and the following couples of primers: 5'TTTGTGCAAACTAGTCGGGAGGAGCAT3' and 5'ATGCTCCTCCCGACTAGTTTGCACAAA3', 5'TTTTCTAAGAAGAGCTCAGAGAGGTGG3' and 5'CCACCTCTCTGAGCTCTTCTTAGAAAA3', 5'TTCTTCCTCTTCTCGAGTGAGTATCGCCCA3' and 5'TGGGCGATACTCACTCGAGAAGAGGAAGAA3'.

HMGB1-ΔC-TM expression and purification

Recombinant HMGB1-ΔC-TM were generated in *Escherichia coli* BL21(DE3)pLysS transformed with pET-22b(pelB) HMGB1-ΔC-TM. Protein expression and purification was performed as described in the Materials and Methods section. The eluted proteins were dialyzed against PBS and then submitted to 12% SDS-PAGE to determine the purity and stored in aliquots at -80°C. The protein concentration was calculated using Bradford's method.

Viability assays

Cell viability was determined by the MTT (methylthiazolyldiphenyl-tetrazolium bromide) assay Briefly, cells were exposed to the indicated concentrations of HGBM1-ΔC or HGBM1-ΔC-TM (C23S-C45S-C106S) for five doubling times and then incubated for 3 hours at 37 °C with MTT. Formazan crystals were then dissolved in dimethylsulfoxide and optical density measured at 570 nm in a microplate reader. The half-maximal inhibitory concentration (IC50) was determined. Results are presented as the percentage of cell viability, determined as the absorbance of the experimental well divided by the absorbance of the untreated control well and the whole multiplied by 100. All values are averages of at least 3 independent experiments done in duplicate.

Immunoblotting

Cells were lysed for 30 minutes at 4°C in lysis buffer (50mM Tris HCl (pH 8.8), 120mM NaCl, 1mM EDTA, 1% NP-40). Proteins were resolved on 12% SDS-PAGE and transferred onto a PVDF membrane (Amersham; GE Healthcare Life Sciences). The membranes were blocked with 5% non-fat dried milk and incubated with the indicated antibodies. Anti-PKM1 and anti-PKM2 antibodies were purchased from Cell Signaling. Primary antibodies were detected by an anti-mouse IgG or anti rabbit IgG, HRP-linked antibodies and immunodetection was achieved with a chemiluminescence reagent (Amersham ECL Prime Western Blotting Detection Reagent). Anti-ß actin antibody (Santa Cruz) was used as a loading control. Immunoblotting were repeated at least twice.

Table S1. Genetic and phenotypic profiles of the cell lines tested.

	Cell line	Phenotypes	Relevant genetic status	MSS vs MSI
Colon	SW48	mesenchymal, invasive	*BRAF* and *KRAS* WT, *TP53* mutant (R248Y)	MSI
	DLD1	epithelial	*KRAS* mutant (G13D), *TP53* mutant (S241F)	MSI
	HT-29	epithelial	*BRAF* mutant (V600E), *TP53* mutant (R273H)	MSS
	HT-29 5FU	mesenchymal, invasive	ND	MSS
	HT-29 oxa	epithelial	ND	MSS
	HT-29 SN-38	epithelial	ND	MSS
	HCT-116	epithelial	*KRAS* mutant (G13D), *TP53* WT	MSI
	LS513	epithelial	*KRAS* mutant (G12D), *TP53* WT	MSS
	LOVO	epithelial	*KRAS* mutant (G13D; A14V), *TP53* WT	MSI
	RKO	mesenchymal, invasive	*BRAF* mutant (V600E), *TP53* WT	MSI
	SW480	mesenchymal, invasive	*KRAS* mutant (G12V), *TP53* mutant (R273H)	MSS
Cervix	HeLa-M	epithelial	*TP53* WT (HPV positive)	
Normal	AB943	fibroblastic		
	C19	fibroblastic		

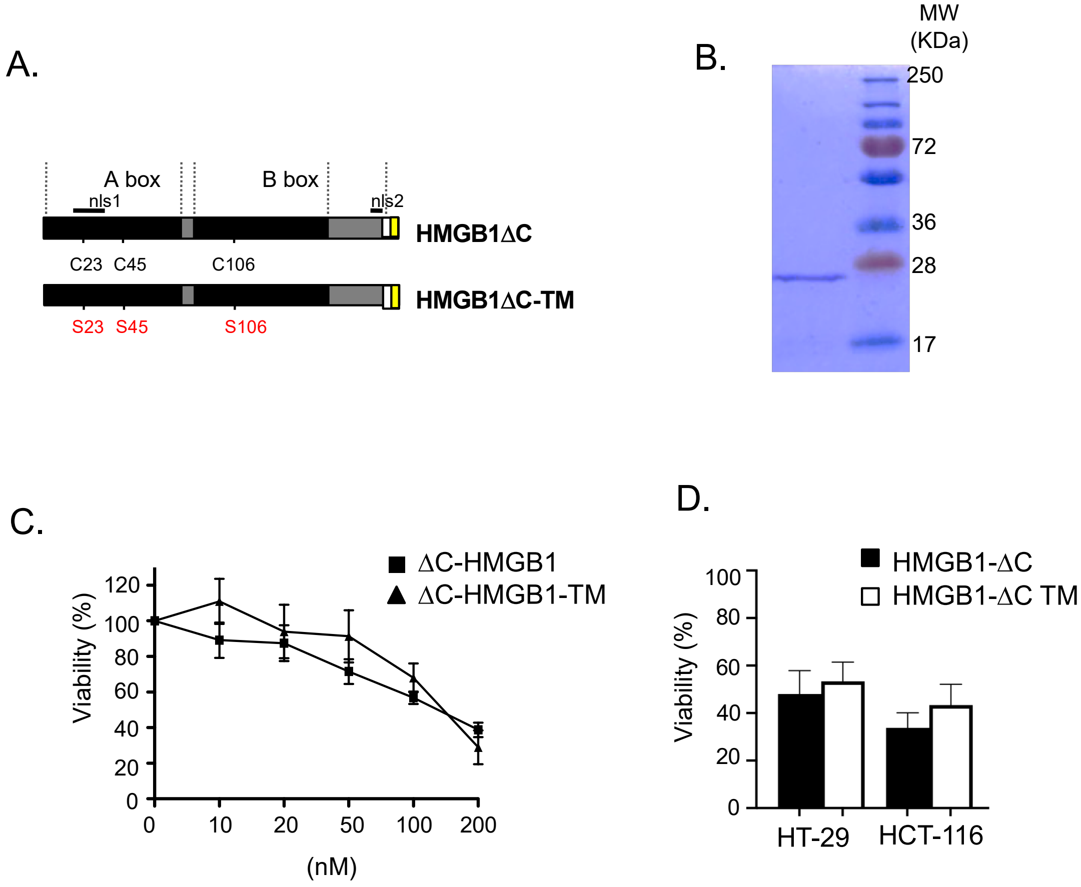

Figure S1. Production and characterization of HMGB1-ΔC-TM. (A) Plasmids construction of HMGB1-ΔC (pET-22b(pelB-)HMGB1-ΔC) and HMGB1-ΔC-TM (pET22b(pelB-)HMGB1-ΔC-TM) are schematically illustrated. Both expression vectors encoded for HMGB1-ΔC and HMGB1-ΔC-TM are fused to a polyhistidine tag at their C-terminus. (B) Expression and purification of HMGB1-ΔC-TM. 1 μg of HMGB1-ΔC-TM was analyzed by SDS-PAGE (12%) after Coomassie blue staining. No ß-mercaptoethanol was added to the loading buffer. (C) HeLa cells were treated for 5 days with increasing concentrations of HMGB1-ΔC and HMGB1-ΔC-TM (range 10-200 nM). Cell viability was assessed by the MTT assay. Standard deviations (SDs) are indicated by error bars when they exceed symbol size. (D) HT-29 and HCT-116 cells were treated for 5 days with 200 nM HMGB1-ΔC and HMGB1-ΔC-TM. Cell viability was assessed by the MTT assay. Standard deviations (SDs) are indicated by error bars.

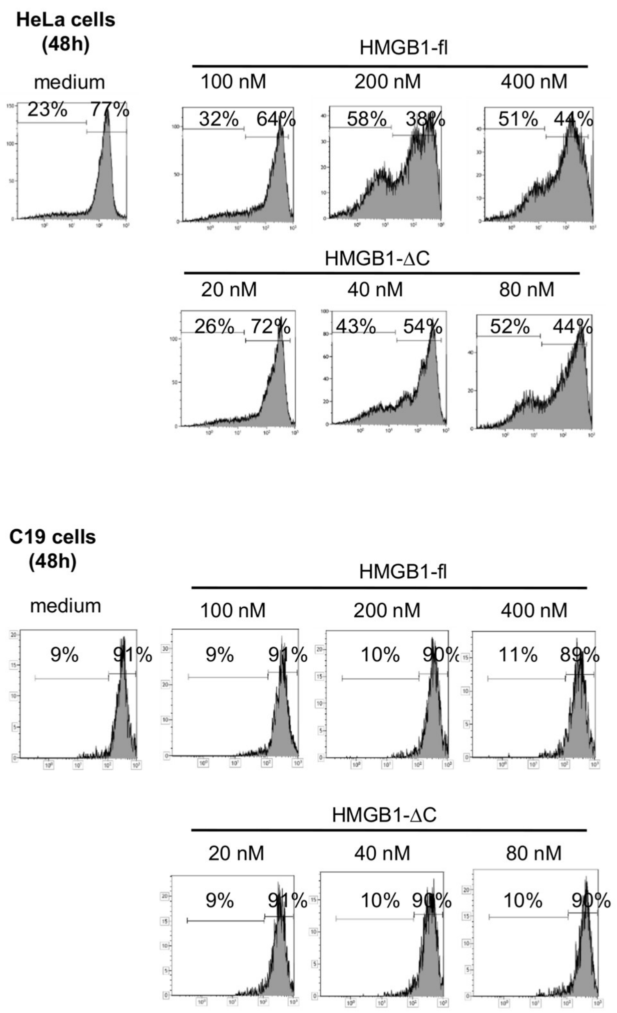

Figure S2. Mitochondrial membrane potential in HeLa (upper panels) and C19 (lower panels) cells after treatment with increasing concentrations of HMGB1-fl (range 100-400 nM) or HMGB1-ΔC (range 20-80 nM). After 48 hours of treatment, cells were labeled for 15 minutes with DIOC6(3) and fluorescence intensities quantified by flow cytometry. The decrease in DIOC6(3) fluorescence corresponds to the loss of $\Delta\psi$m.

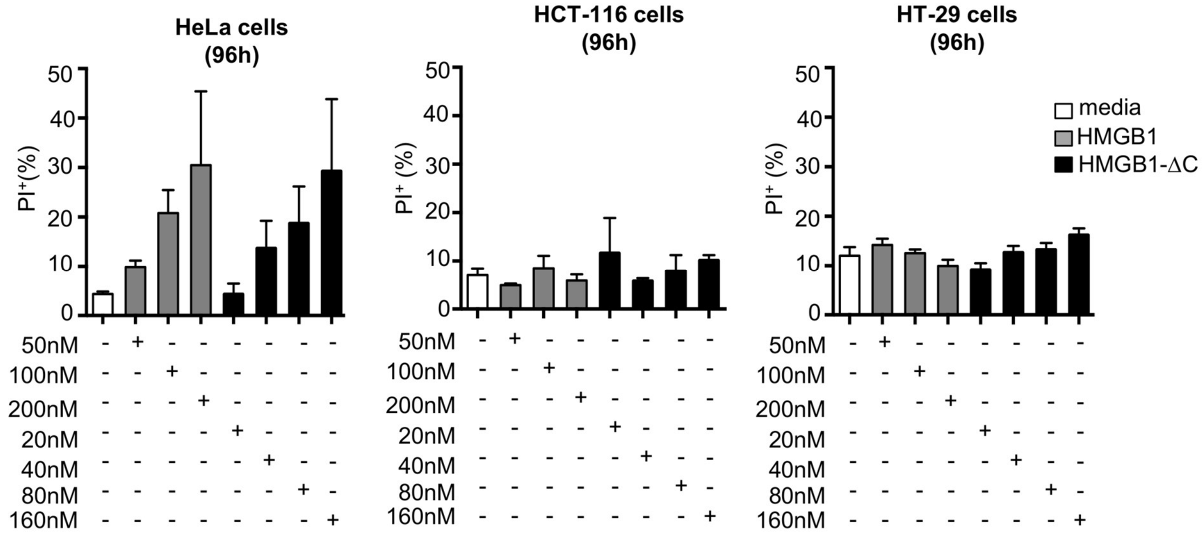

Figure S3. Cell death induction in cells treated with HMGB1-fl or HMGB1-ΔC. The HeLa, HCT-116 and HT-29 cells were treated for 96 hours with increasing concentrations of HMGB1-fl (range 50-200 nM) or HMGB1-ΔC (range 20-160 nM) and labeled with PI. The percentage of dead cells (PI+) was determined by flow cytometry. Data are expressed as mean +/- SD.

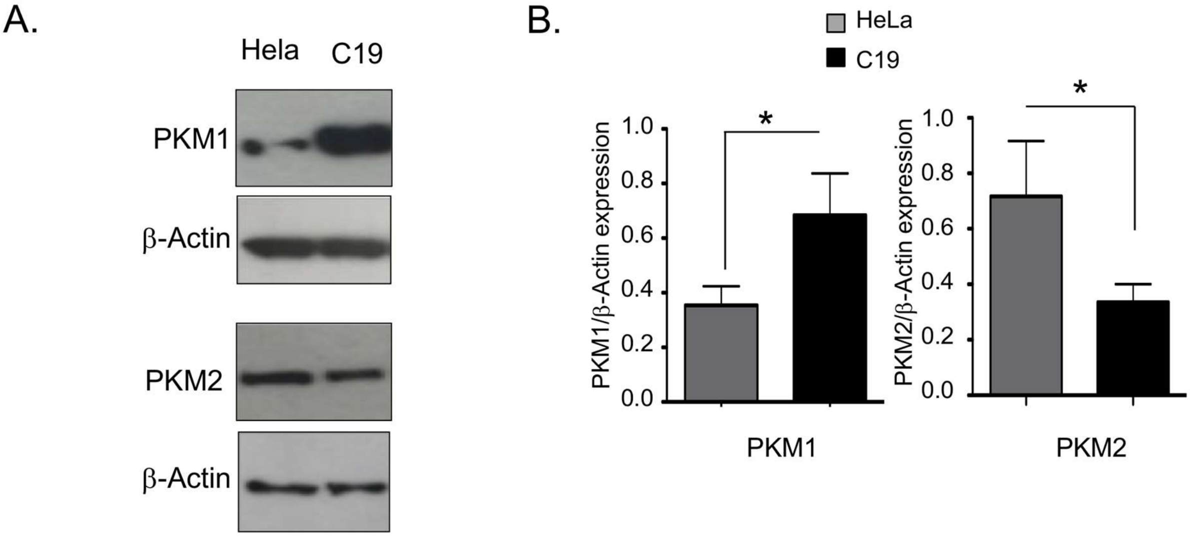

Figure S4. PKM1/2 protein expression profiles in HeLa and C19 cells. PKM1 and PKM2 protein expression levels were determined by immunoblotting the two isoforms in HeLa and C19 cellular extracts (representative image shown in A). (B) Band intensities of 2 independent immunoblots were quantified and normalized with β-actin. Data are expressed as mean +/- SD. *P* values were calculated using the Student paired t test. *$p<0.05$.

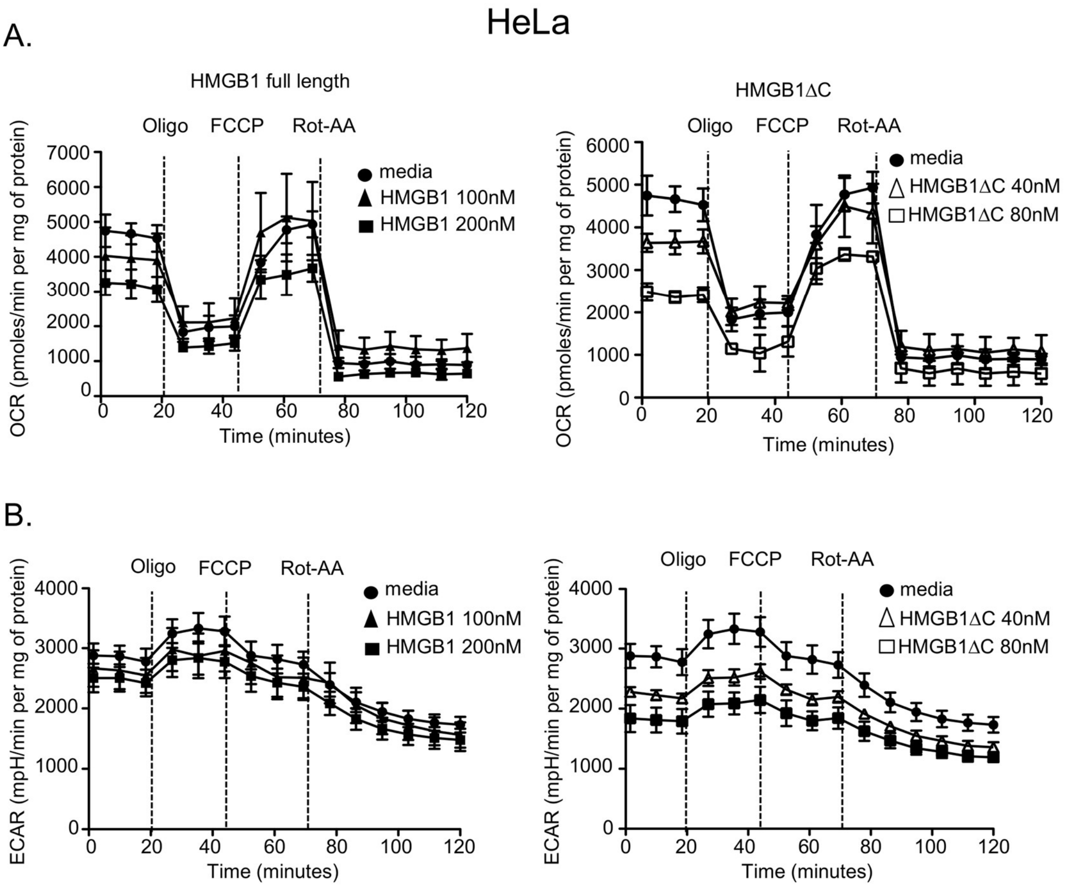

Figure S5. Acute effects of increasing concentrations of HMGB1-fl and HMGB1-ΔC on HeLa cells bioenergetics. (A) HeLa cells were seeded in a Seahorse XFe24 plate, treated for 48 hours with HMGB1-fl (left panel) or HMGB1-ΔC (right panel) and basal OCR measurements were made followed by injection of oligomycin (1 μM), FCCP (1 μM) and rotenone (10 μM) + antimycin A (10 μM). OCR values were normalized with the total amount of proteins present in each well and are expressed in pmoles per min per mg of protein. The data shown are representative of 3 independent experiments. Data are expressed as mean +/- SD. (B) Simultaneously to OCR measurements, basal ECAR measurements were made on HMGB1-fl (left panel) or HMGB1-ΔC (right panel) treated HeLa cells followed by injection of oligomycin (1 μM), FCCP (1 μM) and rotenone (10 μM) + antimycin A (10 μM). ECAR values were normalized with the total amount of proteins present in each well and are expressed in mpH per min per mg of protein. The data shown are representative of 3 independent experiments. Data are expressed as mean +/- SD.

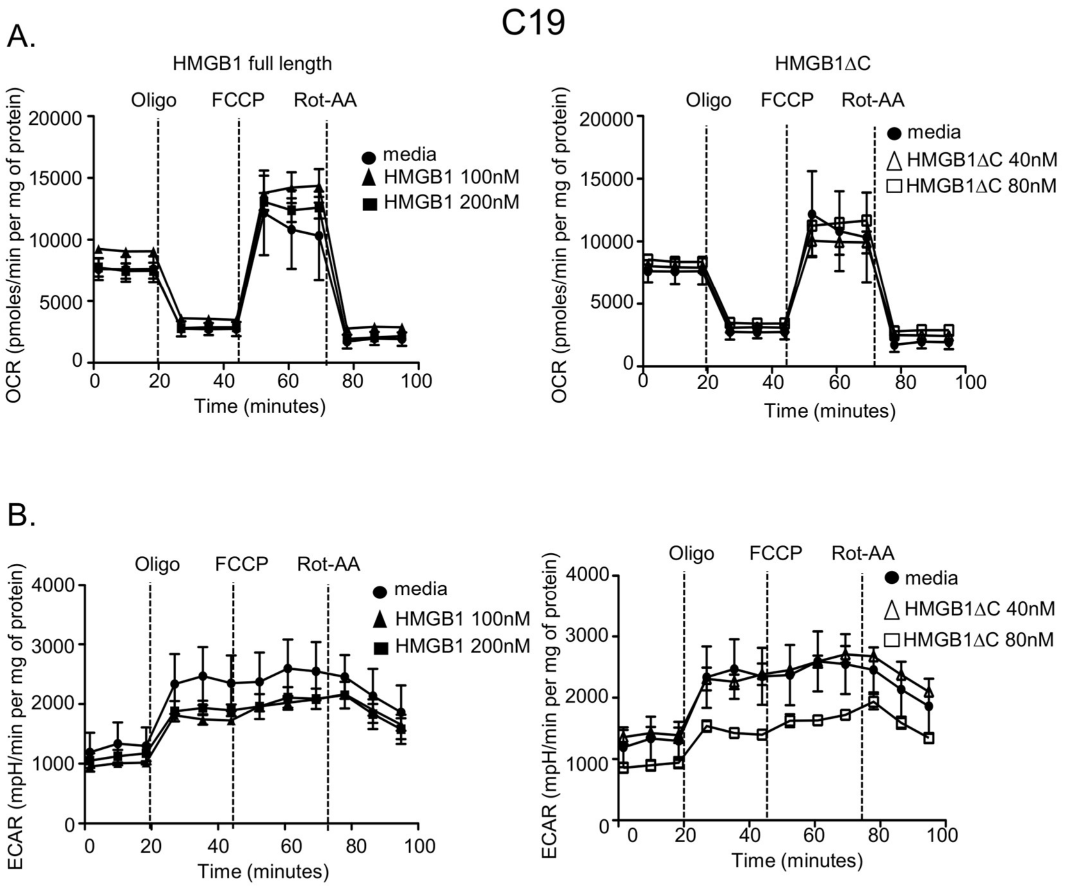

Figure S6. Acute effects of increasing concentrations of HMGB1-fl and HMGB1-ΔC on C19 cells bioenergetics. (A) C19 cells were seeded in a Seahorse XFe24 plate, treated for 48 hours with HMGB1-fl (left panel) or HMGB1-ΔC (right panel) and basal OCR measurements were made followed by injection of oligomycin (1 μM), FCCP (1 μM) and rotenone (10 μM) + antimycin A (10 μM). OCR values were normalized with the total amount of proteins present in each well and are expressed in pmoles per min per mg of protein. The data shown are representative of 3 independent experiments. Data are expressed as mean +/- SD. (B) Simultaneously to OCR measurements, basal ECAR measurements were made on HMGB1-fl (left panel) or HMGB1-ΔC (right panel) treated C19 cells followed by injection of oligomycin (1 μM), FCCP (1 μM) and rotenone (10 μM) + antimycin A (10 μM). ECAR values were normalized with the total amount of proteins present in each well and are expressed in mpH per min per mg of protein. The data shown are representative of 3 independent experiments. Data are expressed as mean +/- SD.

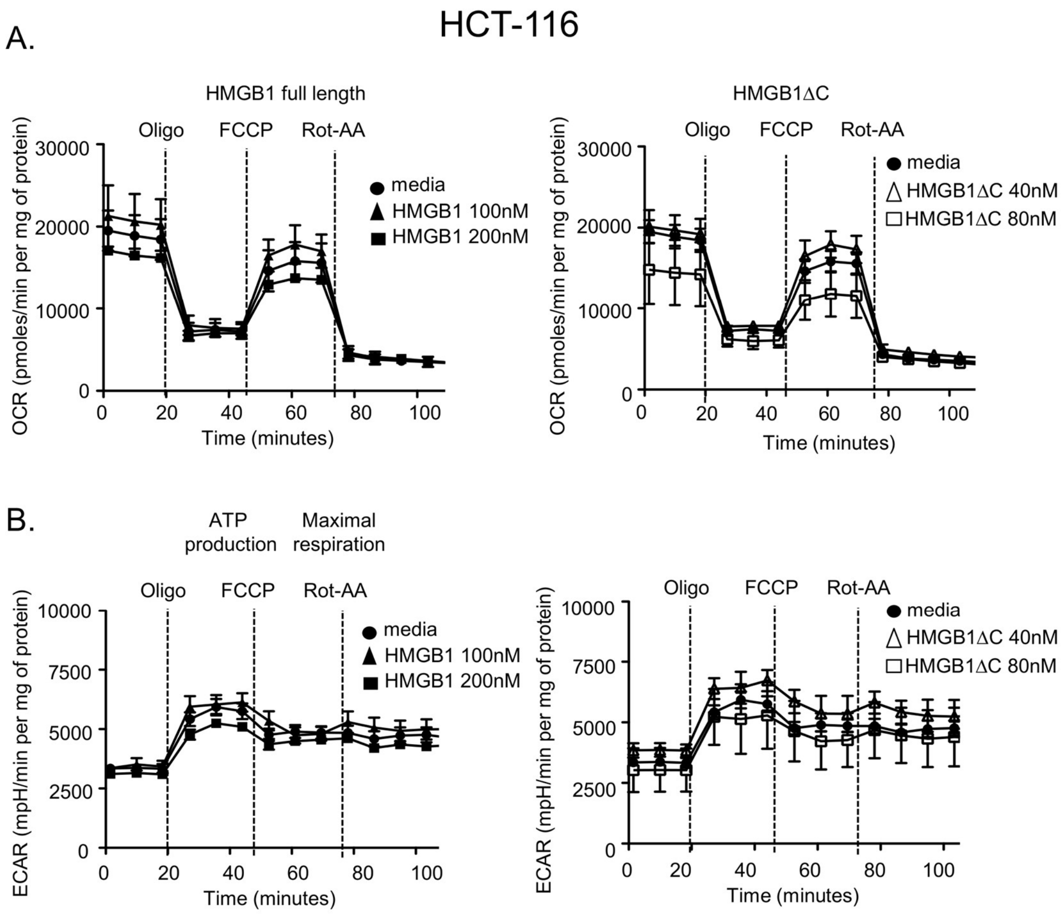

Figure S7 Acute effects of increasing concentrations of HMGB1-fl and HMGB1-ΔC on HCT-116 cells bioenergetics. (A) HCT-116 cells were seeded in a Seahorse XFe24 plate, treated for 48 hours with HMGB1-fl (left panel) or HMGB1-ΔC (right panel) and basal OCR measurements were made followed by injection of oligomycin (1 μM), FCCP (1 μM) and rotenone (10 μM) + antimycin A (10 μM). OCR values were normalized with the total amount of proteins present in each well and are expressed in pmoles per min per mg of protein. The data shown are representative of 3 independent experiments. Data are expressed as mean +/- SD. (B) Simultaneously to OCR measurements, basal ECAR measurements were made on HMGB1-fl (left panel) or HMGB1-ΔC (right panel) treated HCT-116 cells followed by injection of oligomycin (1 μM), FCCP (1 μM) and rotenone (10 μM) + antimycin A (10 μM). ECAR values were normalized with the total amount of proteins present in each well and are expressed in mpH per min per mg of protein. The data shown are representative of 3 independent experiments. Data are expressed as mean +/- SD.

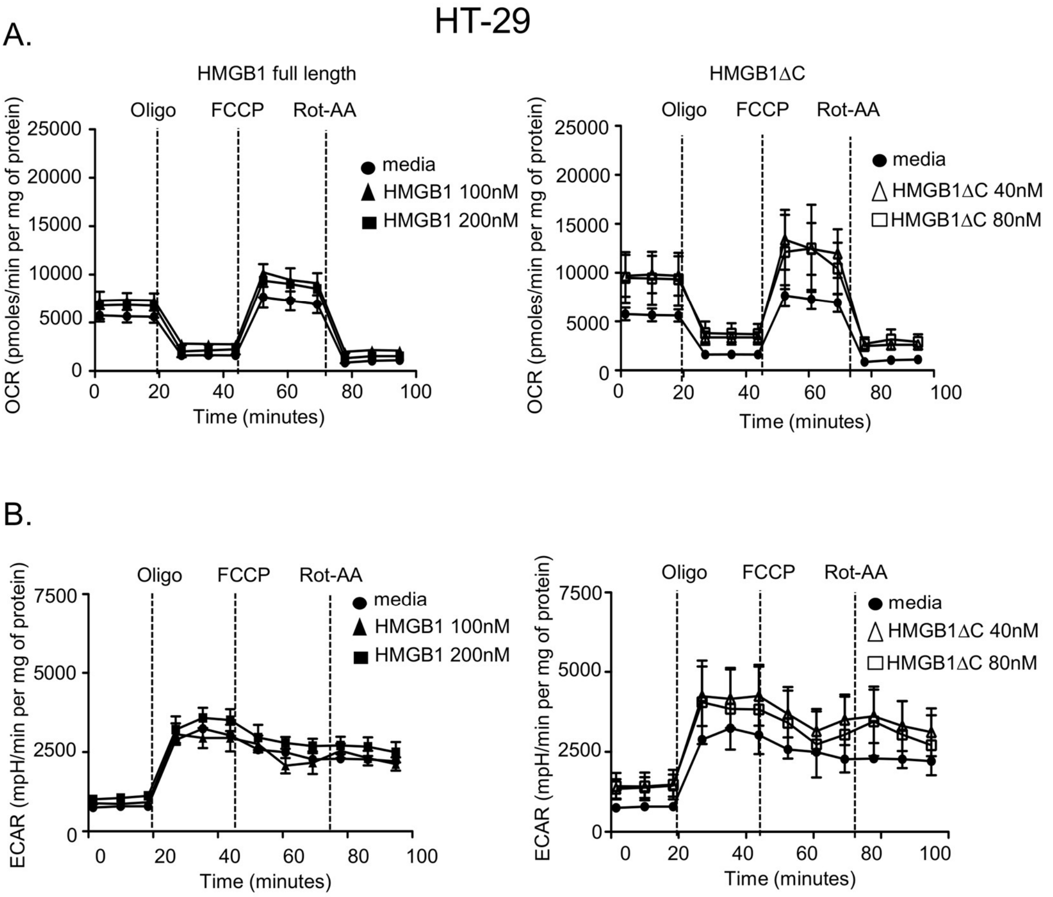

Figure S8. Acute effects of increasing concentrations of HMGB1-fl and HMGB1-ΔC on HT-29 cells bioenergetics. (A) HT-29 cells were seeded in a Seahorse XFe24 plate, treated for 48 hours with HMGB1-fl (left panel) or HMGB1-ΔC (right panel) and basal OCR measurements were made followed by injection of oligomycin (1 μM), FCCP (1 μM) and rotenone (10 μM) + antimycin A (10 μM). OCR values were normalized with the total amount of proteins present in each well and are expressed in pmoles per min per mg of protein. The data shown are representative of 3 independent experiments. Data are expressed as mean +/- SD. (B) Simultaneously to OCR measurements, basal ECAR measurements were made on HMGB1-fl (left panel) or HMGB1-ΔC (right panel) treated HT-29 cells followed by injection of oligomycin (1 μM), FCCP (1 μM) and rotenone (10 μM) + antimycin A (10 μM). ECAR values were normalized with the total amount of proteins present in each well and are expressed in mpH per min per mg of protein. The data shown are representative of 3 independent experiments. Data are expressed as mean +/- SD.
